# Impact factors of *Blastocystis hominis* infection in persons living with human immunodeficiency virus: a large-scale, multi-center observational study from China

**DOI:** 10.1186/s40249-023-01137-5

**Published:** 2023-09-11

**Authors:** Shun-Xian Zhang, Ji-Chun Wang, Zhong-Wei Li, Jin-Xin Zheng, Wen-Ting Zhou, Guo-Bing Yang, Ying-Fang Yu, Xiu-Ping Wu, Shan Lv, Qin Liu, Mu-Xin Chen, Yan Lu, Zhi-Hui Dou, Da-Wei Zhang, Wen-Wen Lv, Lei Wang, Zhen-Hui Lu, Ming Yang, Pei-Yong Zheng, Yue-Lai Chen, Li-Guang Tian, Xiao-Nong Zhou

**Affiliations:** 1 Longhua Hospital, Shanghai University of Traditional Chinese Medicine, Shanghai, 200032 China; 2 Chinese Center for Disease Control and Prevention (Chinese Center for Tropical Diseases Research), NHC Key Laboratory of Parasite and Vector Biology, WHO Collaborating Centre for Tropical Diseases, National Center for International Research On Tropical Diseases, National Institute of Parasitic Diseases, Shanghai, 200025 China; 3 School of Global Health, Chinese Center for Tropical Diseases Research-Shanghai Jiao Tong University School of Medicine, Shanghai, 200025 China; 4 Department of Science and Technology, Chinese Center for Disease Control and Prevention, Beijing, 102206 China; 5 Gansu Province People’s Hospital, Gansu Provincial Hospital, Lanzhou, 730000 China; 6 National Health Commission (NHC) Key Laboratory of Biosafety, National Institute for Viral Disease Control and Prevention, Chinese Center for Disease Control and Prevention, Beijing, 102206 China; 7 Gansu Provincial Center for Disease Control and Prevention, Lanzhou, 730000 China; 8 National Center for AIDS/STD Control and Prevention, China Center for Disease Control and Prevention, Beijing, 102206 China; 9 The People’s Liberation Army 302 Hospital, Beijing, 100039 China; 10 Clinical Research Institute, Shanghai Jiao Tong University School of Medicine, Shanghai, 200025 China

**Keywords:** *Blastocystis hominis*, Human immunodeficiency virus, Viral load, Non-linear association, Restricted cubic spline, Propensity score, China

## Abstract

**Background:**

*Blastocystis hominis* (Bh) is zoonotic parasitic pathogen with a high prevalent globally, causing opportunistic infections and diarrhea disease. Human immunodeficiency virus (HIV) infection disrupts the immune system by depleting CD4^+^ T lymphocyte (CD4^+^ T) cell counts, thereby increasing Bh infection risk among persons living with HIV (PLWH). However, the precise association between Bh infection risk and HIV-related biological markers and treatment processes remains poorly understood. Hence, the purpose of the study was to explore the association between Bh infection risk and CD4^+^ T cell counts, HIV viral load (VL), and duration of interruption in antiviral therapy among PLWH.

**Methods:**

A large-scale multi-center cross-sectional study was conducted in China from June 2020 to December 2022. The genetic presence of Bh in fecal samples was detected by real-time fluorescence quantitative polymerase chain reaction, the CD4^+^ T cell counts in venous blood was measured using flowcytometry, and the HIV VL in serum was quantified using fluorescence-based instruments. Restricted cubic spline (RCS) was applied to assess the non-linear association between Bh infection risk and CD4^+^ T cell counts, HIV VL, and duration of interruption in highly active antiretroviral therapy (HARRT).

**Results:**

A total of 1245 PLWH were enrolled in the study, the average age of PLWH was 43 years [interquartile range (*IQR*): 33, 52], with 452 (36.3%) being female, 50.4% (*n* = 628) had no immunosuppression (CD4^+^ T cell counts > 500 cells/μl), and 78.1% (*n* = 972) achieved full virological suppression (HIV VL < 50 copies/ml). Approximately 10.5% (*n* = 131) of PLWH had interruption. The prevalence of Bh was found to be 4.9% [95% confidence interval (*CI*): 3.8–6.4%] among PLWH. Significant nonlinear associations were observed between the Bh infection risk and CD4^+^ T cell counts (*P*_for nonlinearity_ < 0.001, L-shaped), HIV VL (*P*_for nonlinearity_ < 0.001, inverted U-shaped), and duration of interruption in HARRT (*P*_for nonlinearity_ < 0.001, inverted U-shaped).

**Conclusions:**

The study revealed that VL was a better predictor of Bh infection than CD4^+^ T cell counts. It is crucial to consider the simultaneous surveillance of HIV VL and CD4^+^ T cell counts in PLWH in the regions with high level of socioeconomic development. The integrated approach can offer more comprehensive and accurate understanding in the aspects of Bh infection and other opportunistic infections, the efficacy of therapeutic drugs, and the assessment of preventive and control strategies.

**Graphical Abstract:**

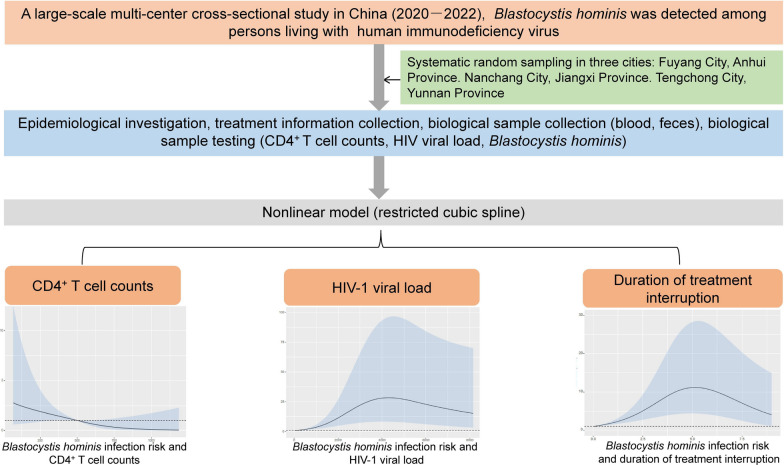

**Supplementary Information:**

The online version contains supplementary material available at 10.1186/s40249-023-01137-5.

## Background

Acquired immune deficiency syndrome (AIDS) is a systemic disease caused by human immunodeficiency virus (HIV) infection [1]. HIV is consisted of two identical negative-sense single-stranded ribonucleic acid (RNA) molecules, belongs to the family *Retroviridae* and the genus *Lentivirus* in virology [2]. HIV is classified into HIV-1 and HIV-2 based on genetic characteristics and differences in viral antigen [3, 4]. HIV-1 is the predominant type in the global as well as China, while HIV-2 is mainly prevalent in West Africa. China is one of the countries with the highest number of persons living with HIV (PLWH) and the most complicated and diversified HIV-1 subtypes, and continues to face significant obstacles and pressures [1, 3]. HIV has significantly impacted global health because of its high transmissibility, high mortality, and lack of effective vaccines and cure drugs [5–7]. Furthermore, the control of this epidemic has been slower than expected. For instance, in 2020, there were 1.5 million new HIV infections globally, with over 650000 AIDS-related deaths [8]. Simultaneously, HIV/AIDS remains a major public health problem and a major cause of deaths in China. At the end of 2020, there were 1.053 million PLWH and 351,000 cumulative reported deaths in China, HIV/AIDS has consistently ranked as the leading cause of death among reported infectious diseases in China in recent years [9, 10]. The coronavirus disease 2019 (COVID-19) had a significant negative impact on HIV/AIDS control efforts globally, it has posed a serious threat to prevention, treatment, and healthcare services for HIV/AIDS individuals. 3.3% had discontinued antiviral therapy (ART) despite tremendous supportive efforts from both governmental and non-governmental organizations, and an estimated only 63.0% new diagnoses were recorded [11]. It is evident that other unforeseen public health emergencies can disrupt the regular ART process for PLWH and contribute to the adverse effects on PLWH health. HIV can lead to a decline in the body's defense function by invading CD4^+^ T lymphocytes (CD4+ T) and macrophages, resulting in various associated diseases. Consequently, PLWH experience opportunistic infections (OPIs) caused by bacteria, viruses, fungus and parasites. These infections are significant contributors to the ultimate mortality [12, 13].

*Blastocystis hominis* (Bh) is a highly prevalent opportunistic pathogen and can be considered a representative of OPIs [14]. Bh is an anaerobic intestinal parasite that can infect human being and various animals through the fecal–oral route, primarily via contaminated water and food sources [12, 15]. The prevalence of Bh ranges from 1.9% to 43.6% in the general population of China, including individuals with diarrhea [12]. Several factors, such as the immune system function, geographic location, age, behaviours of the host, the degree of close contact with livestock and pets, as well as climatic conditions, can impact the occurrence and transmission of Bh [16, 17]. Bh is also prevalent among PLWH [18, 19], and the prevalence of Bh among HIV/AIDS individuals ranges from 30.0% to 47.6% in developed countries [20, 21], the incidence ranges from 3.7% to 20.7% among HIV/AIDS subjects in middle- and low-income countries [12, 19]. Clinical symptoms of Bh infection vary and are generally self-limiting. Mild infections may be asymptomatic carriers with a low parasite count in the stool, while individuals with compromised immune systems may experience gastrointestinal symptoms such as diarrhea and abdominal pain [12]. However, when PLWH infected with Bh, it can lead to severe diarrhea, accelerating the progression of HIV/AIDS and even resulting in death [22].

Prior to the widespread use of highly active antiretroviral therapy (HAART), OPIs are the leading cause of death among PLWH [23]. Widespread use of HAART after 1995 resulted in a marked reduction in deaths among persons with HIV/AIDS, primarily because of reductions in deaths attributable to OPIs [24]. In recent years, the strategy of initiating immediate treatment for PLWH upon diagnosis has been vigorously promoted and implemented on a global scale. This marks the onset of a new era in ART worldwide [25]. The National Free Antiretroviral Therapy Program has been scaled-up since 2003 in China, resulting in a remarkable reduction AIDS-related morbidity and mortality [26, 27]. Meanwhile, the ‘treat all’ strategy was implemented in 2016 in China. Viral replication within patients' bodies is suppressed by initiating HAART, leading to an increase in the CD4^+^ T cell counts and the restoration of immune function. This approach has had a profound impact on reducing mortality associated with OPIs among PLWH [19, 27, 28]. However, certain PLWH who have been prescribed HAART may fail to achieve sufficient virological and immunological responses due to factors related to adherence, pharmacokinetics, or unknown biological factors [26]. Therefore, despite a decrease in hospitalizations and deaths since the implementation of HAART, OPIs remain a major cause of disease progression and mortality among PLWH. Clinical healthcare providers must have a thorough understanding of the optimal strategies for prevention and management of OPIs in order to deliver comprehensive and high-quality healthcare services to HIV/AIDS individuals.

It is well-known that long-term administration of HARRT in PLWH can reduce the OPIs. However, it remains unclear whether the Bh infection upon discontinuation of HARRT is constant or variable. Additionally, the specific association between the Bh infection risk and the duration of interruption in HARRT, immune status and viral replication level is not well understood. Is the association constant or does it exhibit a nonlinear pattern? The aim of the study is to explore the strength of the association between Bh infection risk and CD4^+^ T cell counts, HIV viral load (VL), and duration of interruption in HARRT. The findings of the study can provide scientific evidence for the OPIs prevention and control in PLWH and offer valuable insights for clinical practice.

## Methods

### Study design and setting

A large-scale multi-center cross-sectional study was conducted in three cities of China from June 2020 to December 2021, including Fuyang City in Anhui Province, Nanchang City in Jiangxi Province, and Tengchong City in Yunnan Province. All PLWH registered in Chinese HIV/AIDS Comprehensive Response Information Management System (CRIMS) of the three cities was selected as potential participants [27], and all potential participants received first-line HAART with free of charge according to the Chinese guidelines for the diagnosis and treatment of PLWH [29]. Participation of the study was voluntary for all PLWH. Furthermore, the study adhered to the guidelines outlined in the Strengthening the Reporting of Observational Studies in Epidemiology (STROBE) [30].

### Sample size

This study was a cross-sectional study, and the prevalence of Bh in the intestinal tract of PLWH was the primary indicator for estimating the sample size, and sample size calculation was performed using the "Confidence Intervals for One Proportion" module from Power Analysis and Sample Size Software (Version 15, NCSS LLC., East Kaysville, Utah, United States). Based on the previous study, the detection rate of Bh was 16.2% in fecal sample of PLWH [18]. Considering confidence levels (1 − ɑ) = 0.95, confidence intervals (*CI*s) formula is Exact (Clopper-pearson),* CI*s width (two sided) is 5.0%, the minimum sample size was 874 individuals. Ultimately, a total of 1245 eligible PLWHs were included in the study.

### Sampling and participants

**Fig. 1:**
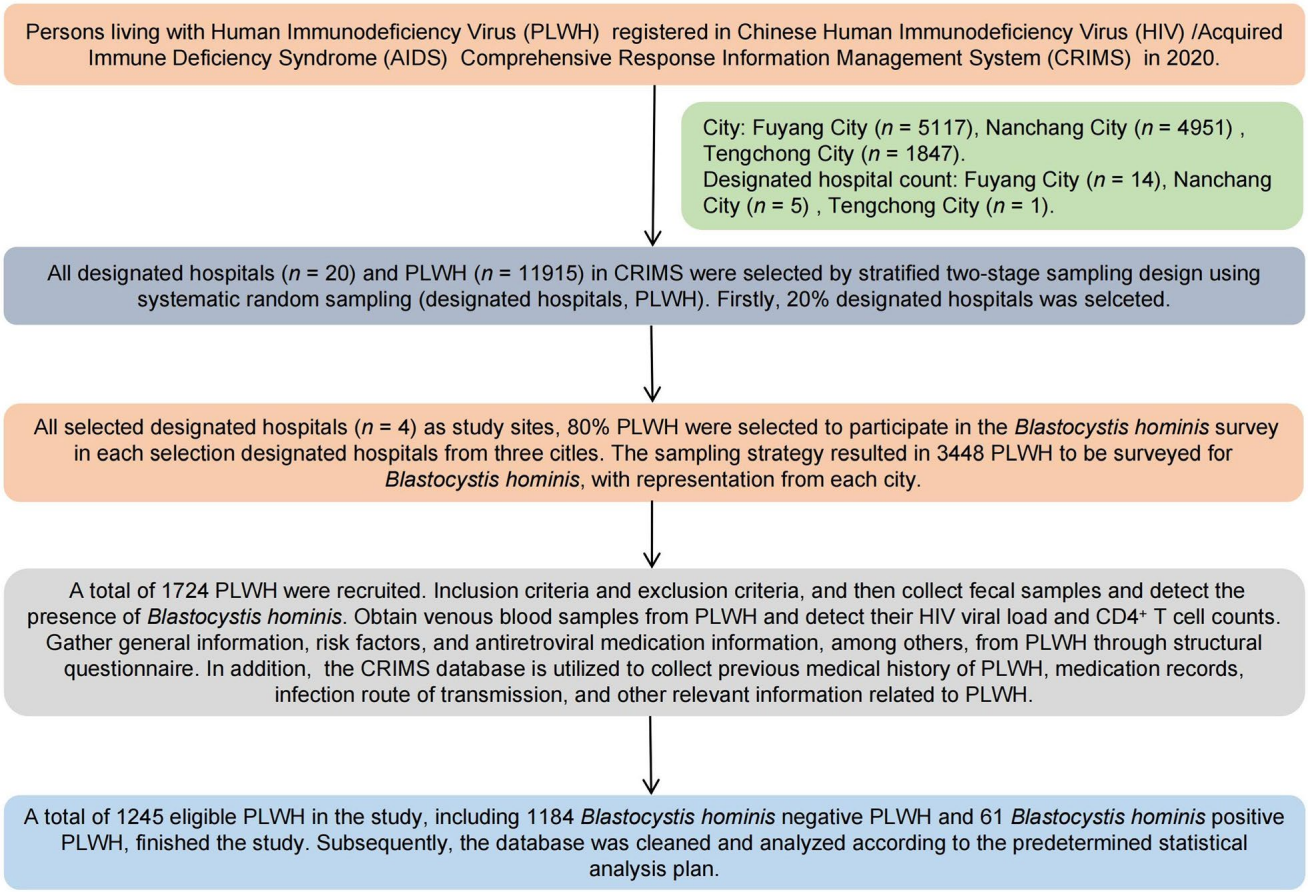
The flowchart of the study

A stratified two-stage sampling approach was employed to select designated hospitals and PLWH in the surveys [31]. According to the CRIMS database, Fuyang City had fourteen county-level designated hospitals, Nanchang City had five, and Tengchong City had one (Fig. 1). The primary sampling frame was the all registered designated hospitals across these cities (stratification factor). A systematic random sampling technique was employed to select one in every five PLWH-designated hospitals. In addition, this selection was adjusted to ensure representation from each city. Specifically, in cities with more than five designated hospitals, a random selection at a 1/5 ratio was conducted. For cities with between one to five designated hospitals, one hospital was chosen at random. If there was only one sentinel hospital available, it represented the exclusive choice. Consequently, this procedure led to the inclusion of four designated hospitals. And four-fifths of PLWH individuals from each designated hospital selected were required to participate in the survey.

For PLWH who had undergone treatment interruption as documented in the National Free Antiretroviral Treatment Program (NFATP) records, their residence were ascertained using the contact details (address, phone number, etc.) in the NFATP records by the staffs from local Center for Disease Control (CDC), and subsequently, they was visited by our study team to gather samples and relevant information. In instances where PLWH had not experienced interruption in HAART, the HIV prevention and control personnel, hailing from either the local CDC or designated hospitals, proactively reached out to these individuals. Subsequently, they were invited to designated hospitals for the purpose of sample and information collection. Furthermore, this study exclusively employed de-identified record data, ensuring that no personally identifiable information was procured. Written informed consent was obtained from all patients or legally authorized representatives. Any study-specific procedures were performed in accordance with all applicable ethical, regulatory, and local requirements. In addition, the data and safety monitoring board oversaw the safety of the study.

PLWH who fulfilled the subsequent inclusion criteria will be eligible for participation: (1) met the diagnostic criteria outlined in the "Diagnostic Criteria for AIDS and HIV Infection (2019 Edition)" issued by the National Health Commission [9, 26]; (2) diagnosed with HIV-1 infection; (3) aged 18 years or older; (4) enrolled in the NFATP between January 1, 2004, and December 31, 2020; (5) PLWH who were alive at the time of the study. (6) Informed consent and voluntarily participated the study. PLWH met one or more of the following criteria will be excluded: (1) PLWH who had incomplete data concerning demographic data, testing results, or other importance information; (2) PLWH who transferred from other regions with unknown treatment and tested information; (3) PLWH who had concurrent immune system disorders or diseases, or had received immunosuppressive therapy; (4) PLWH who resumed anti-HIV treatment after interruption.

### Bh infection diagnosis

Fecal specimens were collected from each PLWH, with sterile containers and stored at − 70 °C at the microbiology laboratory of Fuyang City CDC, Nanchang City CDC and Tengchong City CDC. And then, each frozen stool specimen was delivered to the laboratory of the National Institute of Parasitic Diseases, Chinese CDC (Shanghai, China), and stored at − 70 °C. Each stool specimen was used for molecular detection, Bh DNA was extracted from approximately 250 mg of each stool sample with the QIAmp DNA Stool Mini Kit (Qiagen, Hilden, Germany) according to the manufacturer’s recommended procedures. Total 200 μl genomic DNA was extracted and stored at − 70 °C until use.

Real-time fluorescence quantitative polymerase chain reaction (qPCR) was applied to detect genome of Bh in extracts of stool specimen. qPCR was carried out using the Bh-specific primer pair BL18SPPF1 (5′-AGTAGTCATACGCTCGTCTCAAA-3′)/BL18SR2PP (5′-TCTTCGTTACCCGTTACTGC-3′) targeting the SSU rRNA coding region as previously described [29, 32]. The primers were synthesized by Shanghai Personalbio Technology Co., Ltd (Shanghai, China), and qPCR amplifications were performed using an ABI 7900HT fast real-time PCR System (Applied Biosystems, Foster City, CA). The qPCR reaction mixture (25 μl total volume) consisted of 12.5 μl 2 × SYBR Select Master Mix (Thermofisher Scientific, Waltham, MA), 2 μl DNA extract, 1 μl each of 10 μmol/L forward primer and reverse primer, and 8.5 μl water. The qPCR conditions consisted of one denaturing cycle at 95 °C for 5 min, 45 cycles were run with 5 s of denaturation at 95 °C, 10 s of annealing at 68 °C, and 15 s of extension at 72 °C. Positive (DNA obtained from Bh ST4 cultures) and negative (DNA matrix replaced by water) qPCR controls were performed. Samples with a cycle threshold (*Ct*) values greater than the *Ct* values of the positive control alone (two-fold diluted) were subjected to repeat testing in a qPCR after a tenfold dilution.

### CD4^+^ T cell counts

The levels of CD4^+^ T cell were detected in accordance with the Guidelines for Quality Assurance of CD4^+^ T cell Detection and the National AIDS Test Specification as follows [29, 33]. Hemolysin was added to peripheral venous blood specimen and incubated in the dark at room temperature for 15 min, and the CD4 fluorescent-marked antibodies (20 µl) and mixed anticoagulants (50 µl) were successively added to the tube for detection, and then incubated for 15 min at room temperature. Finally, the absolute counts of CD4^+^ T cell counts were measured using FACS Calibur Flow Cytometry System (BD Company, Franklin Lakes, New Jersey, USA).

### HIV-1 VL

Ethylenediaminetetraacetic acid dipotassium salt dihydrate (EDTA-K2) anticoagulants were taken and centrifuged for 10 min at 1300 × *g* to separate the plasma. The separated plasma was stored in the refrigerator at − 70 °C for standby application. Plasma (1100 µl) was taken from each sample and the HIV RNA content in the plasma samples was detected by reverse transcriptase-polymerase chain reaction in automated qPCR-based NucliSENS ECL (BioMerieux, Marcyl’Etoile, France) with NucliSens HIV-1 QT Amplification kit (BioMerieux, Marcyl'Etoile, France). During the operation, an internal standard for quantification, a strong positive, a weak positive and a negative control were set, respectively.

### Exposure variables

The treatment of PLWH must be continuous without interruption in essence. Based on real medical scene, as supported by experience and literature, PLWH who faithfully adhere to antiviral treatment will encounter a resurgence in HIV VL if the treatment is halted for more than 14 days [34, 35]. As a result, treatment interruption for PLWH refers to the deliberate discontinuation of HAART for a duration of 14 days or more, for various reasons during the clinical study. Duration of interruption in HARRT is defined as the difference between the date of treatment interruption and the date of entry into study, it is one continuous exposure variable in the study. As a result, all PLWH were divided into two groups, the treatment interruption PLWH and the non-treatment interruption PLWH. In addition, both CD4^+^ T cell counts and HIV VL are important biological indicators that reflect the disease progression and the Bh emergence risk. Therefore, CD4^+^ T cell counts and HIV VL were considered as an exposure factors.

### Covariates

Following informed consent, each PLWH was interviewed by a trained interviewer using a standardized questionnaire to collect the following information: gender, height, weight, body mass index (BMI), region, occupation, nationality, marital status, education level, household size, toilet type, water supply, drinking water, livestock (horses, cows, dogs, pigs, chickens, ducks, geese, etc.), pet animal (dogs, cats, rabbits, hamsters, etc.). In the CRIMS database, variables such as HIV infection route, date of diagnosis, date of verification, medical treatments, and baseline information were extracted. Age was calculated based on the difference between the date of study participation and the date of birth for each PLWH.

A standardized protocol was established to ensure uniformity in interviewer training and quality control supervision across all survey instances. Initially, stringent adherence to defined inclusion and exclusion criteria governed the selection of study participants. Furthermore, investigators underwent a preliminary assessment before the official investigation to verify their adeptness in communication skills. Dual data entry was executed by distinct personnel employing REDCap software (version 10.0, Vanderbilt University, Nashville, USA), with cross-validation procedures conducted prior to finalization. Each questionnaire underwent a rigorous review by quality supervision staffs, a thorough examination of the questionnaires was initiated when inconsistencies were found, subsequently, participants were promptly contacted to check information and provide clarifications.

### Definitions

HIV infection time refers to the duration between the date when participant entered the study and the date of HIV diagnosis. HIV/AIDS individual with a CD4^+^ T cell counts greater than 500 cells/μl and HIV infection duration of less than 2 years were defined as newly infection. CD4^+^ T cell staging was primarily based on the diagnostic criteria for HIV/AIDS (WS293–2019) [26, 29]. And the CD4^+^ T cell counts were categorized into four groups (categorical variable) and defined as CD4^+^ T immunological status: ≥ 500 cells/μl as the no immunosuppression, 350 cells/μl ≤ CD4^+^ T cell counts < 500 cells/μl as the mild immunosuppression, 200 cells/μl ≤ CD4^+^ T cell counts < 350 cells/μl as the moderate immunosuppression, and CD4^+^ T cell counts < 200 cells/μl as the severe immunosuppression [29]. Virological status was defined as follows: full virological suppression (VL < 50 copies/ml), low level viraemia (VL between 51 and 999 copies/ml), virological failure (VL > 1000 copies/ml) [29].

### Statistical analyses

Missing data was imputed using a technique known as multiple imputation (MI, R package MICE, R Foundation for Statistical Computing, Vienna, Austria, https://cran.r-project.org/. 20 imputed datasets), which accounts for the uncertainty in the generated values through the addition of randomness during the imputation procedure.

For quantitative data, the normality assumption diagnosis was conducted using the Kolmogorov–Smirnov (KS) test with Lilliefors correction significance. The KS test with a *P*-value < 0.05 was used to determine non-normally distributed variables (including age, BMI, HIV infection time, duration of interruption in HARRT, CD4^+^ T cell counts, HIV VL) in PLWH individuals with or without Bh infection, and were compared using the *Z* test. Non-normally distributed variables were described with median and inter-quartile ranges (IQR*s*) and compared using the *Z* test between Bh positive individuals and Bh negative volunteers. For categorical variables (gender, residence, region, occupation, nationality, marital status, education level, household size, toilet type, water supply, drinking water, livestock, pet, HIV infection route, newly infection, CD4^+^ T immunological status, HIV virological status, treatment interruption), frequency and percentage were used to describe these values. The univariate analysis for the differences between the categorical variable mentioned above and Bh infection or not infection among PLWH was performed using either the chi-square test or Fisher's exact test. Odds ratio (*OR*) and 95% *CI*s of categorical variables were calculated using two tailed. Correlation analyses were carried out using Spearman's correlation or Pearson's correlation to explore the association among age, BMI, duration of interruption in HARRT, CD4^+^ T cell counts, HIV VL and HIV infection time, based on the normality of these variables and the equality of variances. Logistic models were used to explore the association between Bh infection and variables such as HIV virological status, CD4^+^ T immunological status, HIV virological status, treatment interruption, etc. Univariable analysis was performed to identify potential predictors of Bh infection. Factors shown to be significant predictors in univariate analysis (*P* < 0.20) were brought forward to a multivariate analysis by a backward stepwise procedure.

Restricted cubic spline (RCS) curve (placed at the 10th, 50th and 90th percentiles) was drawn in the logistic regression model to assess the potential association between Bh infection and age, BMI, HIV infection time, duration of interruption in HARRT, CD4^+^ T cells counts, HIV VL, respectively. In addition, two methods were used to assess the association between Bh infection and CD4^+^ T cell counts. The first method was propensity score matching (PSM) [36, 37], it can adjust for potential confounding factors, including gender, age, BMI, nationality, residence, region, occupation, marital status, education level, family numbers, toilet type, water supply, drinking water, livestock, pet, HIV infection route, HIV VL, HIV infection time, and duration of interruption in HARRT. Propensity score (PS) was calculated using nearest neighbor matching (at ratios of 1:1, 1:2, and 1:4) based on the logit of the propensity score, with caliper widths of 0.01, 0.02, 0.10, and 0.20. This matching was performed without replacement, and a conditional logistic regression model was used to analyze the data, exploring the relationship between Bh infection and CD4^+^ T cell immunological status. Additionally, multiple models were employed to explore the association, including a multivariate analysis using logistic regression model of RCS with five knots (10th, 50th and 90th percentiles of CD4^+^ T cell counts) following Harrell's recommendations [38, 39], different sets of predictors were employed in multiple models to evaluate the stability of the results in the aforementioned statistical models. Base model (Model 1) only included CD4^+^ T cell counts. Model 2 was adjusted for gender, age, BMI, nationality, residence, region, occupation, marital status, education level, family numbers, toilet type, water supply, drinking water, livestock, pet. Model 3 further adjusted for HIV infection route, HIV VL, HIV infection time, duration of treatment interruption in HARRT.

Similarly, the association between Bh infection and HIV VL was explored using similar methods. The first approach employed was PSM [36–39]. Other variables were balanced initially between PLWH infected with Bh and PLWH not infected with Bh, including gender, age, BMI, nationality, residence, region, occupation, marital status, education level, family size, toilet type, water supply, drinking water source, livestock ownership, pet ownership, HIV infection route, CD4^+^ T cell counts, duration of interruption in HARRT, HIV infection time. PS were calculated and matching was performed using nearest neighbor matching (1:1, 1:2, 1:4) based on the logit of the propensity scores, with a caliper width of 0.01, 0.02, 0.10, 0.20, without replacement. Subsequently, a conditional logistic regression model was used to analyze the data and investigate the relationship between Bh infection and HIV virological status. Additionally, nonlinear models was constructed [40]. The baseline model (Model 4) included only HIV VL. Model 5 was adjusted for gender, age, BMI, nationality, residence, region, occupation, marital status, education level, family size, toilet type, water supply, drinking water source, livestock ownership, and pet ownership. Model 6 further adjusted for HIV infection route, CD4^+^ T cell counts, HIV infection time, and duration of interruption in HARRT.

The association between Bh infection and duration of interruption in HARRT was explored using similar methods. Firstly, PSM was employed to adjust for confounding factors [36], it involved balancing other variables between PLWH individuals with and without Bh infection, including gender, age, BMI, nationality, residence, region, occupation, marital status, education level, family size, toilet type, water supply, drinking water source, livestock ownership, pet ownership, HIV infection route, CD4^+^ T cell counts, HIV VL and HIV infection time. The PS was calculated using the logit of the propensity score and matched using nearest neighbor matching (1:1, 1:2, 1:4) with a caliper width of 0.01, 0.02, 0.10, and 0.20. The matching was performed without replacement. Subsequently, a conditional logistic regression model was employed to analyze the data and explore the association between Bh infection and treatment interruption. Additionally, nonlinear models were constructed [40]. The baseline model (Model 7) included only duration of interruption in HARRT. Model 8 was adjusted for gender, age, BMI, nationality, residence, region, occupation, marital status, education level, family size, toilet type, water supply, drinking water source, livestock ownership, and pet ownership. Model 9 further adjusted for HIV infection route, CD4^+^ T cell counts, HIV VL and HIV infection time.

All statistical analyses were performed using R software version 4.2.3 (R Foundation for Statistical Computing, Vienna, Austria, https://cran.r-project.org/). All statistical tests were two-tailed, and the significance level was set at *P* < 0.05.

### Sensitivity analysis

To validate the robustness of the main findings, a sensitivity analysis was conducted to address the following question: to what extent is CD4^+^ T immunological status, HIV virological status, and treatment interruption, associated with Bh infection after controlling for all confounding factors? In other words, how strong would any uncontrolled confounding factors have to be to overturn our statistically significant results? Sensitivity analyses for measured and unmeasured confounding were performed using the full model (model 3, model 6, model 9, respectively).

The first approach aimed to address unmeasured confounding factors. E-value sensitivity analysis was used to quantify the potential implications of unmeasured confounders and found that an unmeasured confounder was unlikely to explain the entirety of the association [41]. E-value is the minimum strength of association, on the risk ratio scale, that an unmeasured confounder would need to have with both the exposure and the outcome to fully explain away a specific exposure-outcome association, conditional on the measured confounding. In order to further address unmeasured confounding [41], the E-value was calculated to estimate the effect of unmeasured confounding factors on the association between the Bh infection and CD4^+^ T immunological status, HIV virological status, and treatment interruption months.

The second approach aimed to address the measured confounding factors by modifying the statistical methodology. The least absolute shrinkage and selection operator (lasso) logistic regression model with penalty parameter tuning was employed to conduct through tenfold cross-validation based on the minimum criteria. The predictive features selected by lasso were then input into binary logistic regression to investigate the potential associations between Bh infection and CD4^+^ T immunological status, HIV virological status, and treatment interruption. The third approach, also targeting the measured confounding factors, involved the utilization of several novel artificial intelligence (AI) methods to construct models. Specifically, the models employed were the Gradient Boosting Machine (GBM), Artificial Neural Network (ANN), Random Forest (RF), and eXtreme Gradient Boosting (XGBOOST) algorithms. Prior to model construction, the entire database was randomly divided into training sets and testing sets at a ratio of 7:3. Due to the limited number of variables, no feature selection was performed in all models, and all variables were included in the several models. The model's predictive performance was evaluated using metrics such as the area under the receiver operating characteristic curve (AUROC), accuracy, precision, sensitivity, specificity, F1 score. Feature importance scores, representing the contribution of predictors to Bh infection, were generated to assess the importance degree of each variable. Higher scores indicated greater relevance to Bh infection. The top 5 features, selected based on their importance scores, were considered the most influential in identifying associations between Bh infection and CD4^+^ T immunological status, HIV virological status, and treatment interruption. If multiple machine learning models consistently indicated that the feature importance scores of CD4^+^ T immunological status, HIV virological status, and treatment interruption ranked highly, it would suggest a strong association between Bh infection and these three factors. All statistical analyses were performed using R software version 4.2.3.

## Results

### Baseline characteristics

A total of 1245 participants of PLWH were enrolled in our study from June 2020 to December 2022 (Fig. 1). The average age of all PLWH was 43 years (*IQR*: 33, 52). The average HIV infection time was 7 years (IQR: 3, 13), and the median CD4^+^ T cell counts was 502 cells/μl (*IQR*: 389, 603). The average HIV VL was 40 copies/ml (IQR: 40, 40) (Additional file 1: Tables S1, S2). The study included 452 (36.3%) female PLWH, and those over 60 years accounted for 11.3% (*n* = 141). There were 94 individuals (7.6%) with low body weight and 8 individuals (0.6%) who were overweight. A total of 382 participants (30.7%) had a family size of three or fewer, while 939 participants (74.2%) resided in rural areas, 877 HIV/AIDS individuals (70.4%) were engaged in farming. The education level of 82 participants (6.6%) was at the college level or above. In terms of sanitation, 385 individuals (30.9%) used non-flush toilets, and 485 individuals (39.0%) did not have access to piped water. Moreover, there were 270 individuals (21.7%) who raised livestock, and 137 individuals (11.0%) had pets. Additionally, 107 participants (8.6%) were newly infected. The majority of PLWH (*n* = 815, 65.5%) acquired HIV through sexual transmission. Approximately 50.4% (*n* = 628) had no immunosuppression (CD4^+^ T cell counts > 500 cells/μl), and the majority (*n* = 972, 78.1%) achieved full virological suppression (HIV VL < 50 copies/ml). Around 10.5% (*n* = 131) experienced interruption in antiretroviral treatment (Table 1). Finally, the prevalence of Bh in the PLWH individuals was 4.9% (95% *CI*: 3.8–6.4%).

**Table 1:** Univariate analysis with logistic regression for *Blastocystis hominis* infection in persons living with human immunodeficiency virus

Variables	Subgroups	Total *n* (%)	*Blastocystis hominis* negative *N* = 1184 *n* (%)	*Blastocystis hominis* positive *N* = 61 *n* (%)	Univariate analysis		
					Wald *χ*^2^	*P* value	*OR *(95% *CI*s)
Age	< 60 years	1104 (88.7)	1051 (88.8)	53 (86.9)	0.204	0.651	1 (reference)
	≥ 60 years	141 (11.3)	133 (11.2)	8 (13.1)			1.193 (0.555–2.563)
BMI	Normal weight (18.5–24.9 kg/m^2^)	1143 (91.8)	1084 (91.6)	59 (96.7)	1.593	0.451	1 (reference)
	Low weight (< 18.5 kg/m^2^)	94 (7.6)	92 (7.8)	2 (3.3)			0.399 (0.096–1.661)
	Over weight (≥ 25.0 kg/m^2^)	8 (0.6)	8 (0.7)	0 (0.0)			–
Family members	< 3 individuals	382 (30.7)	368 (31.1)	14 (23.0)	1.780	0.182	1 (reference)
	≥ 3 individuals	863 (69.3)	816 (68.9)	47 (77.0)			1.514 (0.823–2.785)
Gender	Male	793 (63.7)	756 (63.9)	37 (60.7)	0.256	0.613	1 (reference)
	Female	452 (36.3)	428 (36.1)	24 (39.3)			1.146 (0.676–1.941)
Residence	Rural area	939 (75.4)	893 (75.4)	46 (75.4)	0.001	0.998	1 (reference)
	Urban area	306 (24.6)	291 (24.6)	15 (24.6)			1.001 (0.551–1.819)
Region	Nanchang city	411 (33.0)	387 (32.7)	24 (39.3)	1.163	0.559	1 (reference)
	Fuyang city	479 (38.5)	458 (38.7)	21 (34.4)			0.739 (0.405–1.349)
	Tengchong city	355 (28.5)	339 (28.6)	16 (22.2)			0.761 (0.398–1.457)
Occupation	Student	50 (4.0)	48 (4.1)	2 (3.3)	3.731	0.292	1 (reference)
	Farmer	877 (70.4)	835 (70.5)	42 (68.9)			1.207 (0.284–5.136)
	Other occupation	195 (15.7)	181 (15.3)	14 (23.0)			1.856 (0.408–8.449)
	Worker	123 (9.9)	120 (10.1)	3 (4.9)			0.600 (0.097–3.704)
Nationality	Han nationality	1207 (96.9)	1148 (97.0)	59 (96.7)	0.011	0.916	1 (reference)
	Minority nationality	38 (3.1)	36 (3.0)	2 (3.3)			1.081 (0.254–4.598)
Marital status	Single	320 (25.7)	307 (25.9)	13 (21.3)	0.645	0.422	1 (reference)
	Married	925 (74.3)	877 (74.1)	48 (78.7)			1.293 (0.691–2.418)
Educational level	High school or below	1163 (93.4)	1105 (93.3)	58 (95.1)	0.288	0.592	1 (reference)
	University or above	82 (6.6)	79 (6.7)	3 (4.9)			0.723 (0.222–2.361)
Toilet type	Flush toilet	860 (69.1)	818 (69.1)	42 (68.9)	0.002	0.969	1 (reference)
	Non-flush toilet	385 (30.9)	366 (30.9)	19 (31.1)			1.011 (0.580–1.762)
Water supply	Tap water	760 (61.0)	721 (60.9)	39 (63.9)	0.225	0.636	1 (reference)
	Non tap water	485 (39.0)	463 (39.1)	22 (36.1)			0.878 (0.514–1.501)
Drinking water	Boiled water	1199 (96.3)	1140 (96.3)	59 (96.7)	0.031	0.861	1 (reference)
	Non-boiled water	46 (3.7)	44 (3.7)	2 (3.3)			0.878 (0.208–3.711)
Livestock	No	975 (78.3)	930 (78.5)	45 (73.8)	0.775	0.379	1 (reference)
	Yes	270 (21.7)	254 (21.5)	16 (26.2)			1.302 (0.724–2.342)
Pet	No	1108 (89.0)	1051 (88.8)	57 (93.4)	1.259	0.262	1 (reference)
	Yes	137 (11.0)	133 (11.2)	4 (6.6)			0.555 (0.198–1.553)
Newly infection	No (≥ 2 years)	1138 (91.4)	1079 (91.1)	59 (96.7)	2.109	0.146	1 (reference)
	Yes (< 2 years)	107 (8.6)	105 (8.9)	2 (3.3)			0.348 (0.084–1.446)
HIV infection route	Sexually transmission	815 (65.5)	778 (65.7)	37 (60.7)	0.685	0.710	1 (reference)
	Transmission among injectors	40 (3.2)	38 (3.2)	2 (3.3)			1.107 (0.257–4.764)
	Blood transmission	390 (31.3)	368 (31.1)	22 (36.1)			1.257 (0.731–2.162)
CD4^+^ T immunological status	No immunosuppression (> 500 cells/μl)	628 (50.4)	625 (52.8)	3 (4.9)	71.388	< 0.001	1 (reference)
	Mild immunosuppression (350–500 cells/μl)	411 (33.0)	396 (33.4)	15 (24.6)			7.891 (2.270–27.433)
	Moderate immunosuppression (200–350 cells/μl)	104 (8.4)	83 (7.0)	21 (34.4)			52.711 (15.389–180.552)
	Severe immunosuppression (≤ 200 cells/μl)	102 (8.2)	80 (6.8)	22 (36.1)			52.711 (15.389–180.552)
HIV virological status	Full virological suppression (< 50 copies/ml)	972 (78.1)	968 (81.8)	4 (6.6)	69.345	< 0.001	1 (reference)
	Low level viraemia (51–999 copies/ml)	92 (7.4)	80 (6.8)	12 (19.7)			36.300 (11.444–115.140)
	Virological failure (> 1000 copies/ml)	181 (14.5)	136 (11.5)	45 (73.8)			80.074 (28.352–226.147)
Treatment interruption	No	1114 (89.5)	1087 (91.8)	27 (44.3)	90.201	< 0.001	1 (reference)
	Yes	131 (10.5)	97 (8.2)	34 (55.7)			14.111 (8.172–24.367)

The “–” symbol indicates that data could not be calculated. BMI: Body mass index. *CI*s: Confidence intervals. HIV: Human immunodeficiency virus. *OR*: Odds ratio

HIV VL, HIV infection time, and duration of interruption in HARRT of PLWH with Bh infection were significantly higher than that of PLWH without Bh infection *(Z* = 14.401, *P* < 0.001. *Z* = 2.414, *P* = 0.016. *Z* = 12.540, *P* < 0.001. *Z* = 2.414,* P* = 0.016). On the other hand, CD4^+^ T cell counts was significantly lower in PLWH with Bh infection compared to those without Bh infection (*Z* = 8.226, *P* < 0.001). Additionally, there were no statistically significant differences in age, BMI, and family members between PLWH with and without Bh infection.

Through Spearman correlation analysis, the results showed that there was a significant negative correlation between duration of interruption in HARRT and CD4^+^ T cell counts (coefficient = − 0.257, *P* < 0.001). Additionally, CD4^+^ T cell counts showed a significant negative correlation with HIV VL (coefficient = − 0.557, *P* < 0.001), while duration of interruption in HARRT exhibited a positive correlation with HIV VL (coefficient = 0.607, *P* < 0.001) (Additional file 1: Table S3). Furthermore, HIV infection time demonstrated a negative correlation with CD4^+^ T cell counts and duration of interruption in HARRT, while it showed a significant positive correlation with CD4^+^ T cell counts (Additional file 1: Table S3).

### Univariate and multivariate analysis for Bh infection in PLWH

Univariate logistic regression analysis revealed no statistical difference in Bh infection between rural and urban inhabitants (*χ*^2^ = 0.001, *P* = 0.998). Similarly, there was no statistical difference in the prevalence of Bh infection among PLWH with different education levels (*χ*^2^ = 0.228, *P* = 0.592), age (< 60 years vs ≥ 60 years, *χ*^2^ = 0.204, *P* = 0.651), different BMI levels (*χ*^2^ = 1.539, *P* = 0.451). Gender (*χ*^2^ = 0.256, *P* = 0.613), nationalities (ethnic Han vs minority groups, *χ*^2^ = 0.011, *P* = 0.916), different marital statuses (*χ*^2^ = 0.645, *P* = 0.422), various occupations (*χ*^2^ = 3.371, *P* = 0.292), and different modes of HIV transmission (*χ*^2^ = 0.685, *P* = 0.710). Raising livestock (*χ*^2^ = 0.775, *P* = 0.528), raising pet (*χ*^2^ = 1.259, *P* = 0.577), different toilet type (flush toilet vs non-flush toilet, *χ*^2^ = 0.002, *P* = 0.929), drinking water (boiled water vs non-boiled water, *χ*^2^ = 0.031, *P* = 0.861), different water supply (tap water vs non tap water, *χ*^2^ = 0.225, *P* = 0.636). Similarly, the chances of *Bh* presence in PLWH with new HIV infection (< 2 years) was as high as those that were taking HIV infection over 2 years (*χ*^2^ = 2.109, *P* = 0.146. Table 1).

However, the chances of Bh infection in PLWH of duration of interruption in HARRT was 14.111 times higher than in HIV/AIDS individuals who consistently took HIV drugs. There was a significant difference in the association between Bh infection and the three levels of HIV virological status (*χ*^2^ = 69.345, *P* < 0.001). Compared to the full virological suppression PLWH, HIV/AIDS with virological failure had the strongest association with Bh infection (*OR* = 80.074, 95% *CI:* 28.352–226.147), followed by low level viraemia (*OR* = 36.330, 95% *CI:* 11.444–115.140). There was a significant statistical difference between the Bh infection and different CD4^+^ T cell counts (*χ*^2^ = 71.388, *P* < 0.001). The Bh infection risk was significantly higher in PLWH with severe immunosuppression, moderate immunosuppression, and mild immunosuppression compared to PLWH without any immunosuppression (*OR* = 52.711, 95% *CI*: 15.387–180.552; *OR* = 52.711, 95% *CI*: 15.389–180.552. *OR* = 7.891, 95% *CI*: 2.270–27.433. Table 1).

Above all, several variables showed potential associations (*P* < 0.20) with Bh infection among PLWH by univariate analysis, including family members, newly infection, CD4^+^ T immunological status, HIV virological status, and treatment interruption. Finally, multivariable analysis revealed that CD4^+^ T immunological status (*χ*^2^ = 8.573, *P* = 0.036), HIV virological status (*χ*^2^ = 23.557, *P* < 0.001), and treatment interruption (*χ*^2^ = 12.055, *P* = 0.001) were identified as independent risk factors for Bh infection. Specifically, compared to PLWH with no immunosuppression, individuals with moderate immunosuppression had a 6.245-fold increase in risk Bh infection, while those with mild immunosuppression had a 366.5% increased of Bh infection risk. Compared to PLWH with full virological suppression, HIV/AIDS individuals with virological failure had a 23.985-fold increase risk of Bh infectio, while those with low level viraemia had a 12.746-fold increase risk of Bh infection. Furthermore, HIV/AIDS individuals who experienced treatment interruption (*OR* = 3.072, 95% *CI:* 1.630–5.788) were more likely to be infected by Bh (Additional file 1: Table S4).

The overall AUROC of the fitted model was 0.907 (95% *CI*: 0.996–0.948. *P* < 0.001). Specifically, the AUROC for treatment interruption was 0.738 (95% *CI*: 0.660–0.815. *P* < 0.001), the AUROC for CD4^+^ T immunological status was 0.841 (95% *CI*: 0.795–0.888. *P* < 0.001), and the AUROC for HIV virological status was 0.890 (95% *CI*: 0.851–0.925, *P* < 0.001).

### PSM found impact factors of Bh infection

PSM analysis was conducted using four different caliper levels (0.01, 0.02, 0.10, 0.20) and three matching ratios (1:1, 1:2, 1:4), while controlling for various confounding factors. There were statistically significant differences in the risk of Bh infection among PLWH with different levels of CD4^+^ T immunological status (all *P* < 0.05). Compared to PLWH with no immunosuppression, the risk of Bh infection was 7.857 to 198.363 times higher in that of the severe immunosuppression HIV/AIDS individuals, 8.090 times to 147.072 times higher in the moderate immunosuppression PLWH, and 2.357 times to 10.579 times higher in the mild immunosuppression ones (Table 2. Additional file 1: Tables S5, S6).

**Table 2:** Propensity score method was used to explore the association between *Blastocystis hominis* infection and CD4^+^ T immunological status, HIV virological status, and treatment interruption

Variables	Subgroups	Matching ratio	Caliper	*Blastocystis hominis* negative *n*	*Blastocystis hominis* positive *n*	Wald *χ*^2^	*P *value	*OR* (95% *CI*s)
CD4^+^ T immunological status^a^	No immunosuppression (> 500 cells/μl)	1:1	0.01	55	55	14.406	0.002	1 (reference)
	Mild immunosuppression (350–500 cells/μl)							4.194 (1.195–14.716)
	Moderate immunosuppression (200–350 cells/μl)							8.095 (2.372–27.623)
	Severe immunosuppression (≤ 200 cells/μl)							7.857 (2.352–26.251)
CD4^+^ T immunological status^a^	No immunosuppression (> 500 cells/μl)	1:1	0.02	57	57	18.868	< 0.001	1 (reference)
	Mild immunosuppression (350–500 cells/μl)							4.206 (1.199–14.759)
	Moderate immunosuppression (200–350 cells/μl)							9.952 (2.945–33.631)
	Severe immunosuppression (≤ 200 cells/μl)							9.308 (2.786–31.097)
CD4^+^ T immunological status^a^	No immunosuppression (> 500 cells/μl)	1:2	0.01	44	88	18.586	< 0.001	1 (reference)
	Mild immunosuppression (350–500 cells/μl)							10.062 (1.218–83.115)
	Moderate immunosuppression (200–350 cells/μl)							147.072 (11.600–1864.698)
	Severe immunosuppression (≤ 200 cells/μl)							115.786 (10.087–1329.131)
CD4^+^ T immunological status^a^	No immunosuppression (> 500 cells/μl)	1:2	0.02	49	98	26.335	< 0.001	1 (reference)
	Mild immunosuppression (350–500 cells/μl)							3.972 (1.108–14.238)
	Moderate immunosuppression (200–350 cells/μl)							13.684 (3.962–47.268)
	Severe immunosuppression (≤ 200 cells/μl)							12.381 (3.679–41.664)
HIV virological status^b^	Full virological suppression (< 50 copies/ml)	1:1	0.01	55	55	16.341	< 0.001	1 (reference)
	Low level viraemia (51–999 copies/ml)							11.062 (1.852–66.073)
	Virological failure (> 1000 copies/ml)							22.191 (4.874–101.031)
HIV virological status^b^	Full virological suppression (< 50 copies/ml)	1:1	0.02	57	57	18.236	< 0.001	1 (reference)
	Low level viraemia (50–999 copies/ml)							12.082 (2.199–66.367)
	Virological failure (> 1000 copies/ml)							24.928 (5.695–109.110)
HIV virological status^b^	Full virological suppression (< 50 copies/ml)	1:2	0.01	47	94	22.522	< 0.001	1 (reference)
	Low level viraemia (51–999 copies/ml)							24.000 (5.186–111.077)
	Virological failure (> 1000 copies/ml)							31.304 (7.537–130.013)
HIV virological status^b^	Full virological suppression (< 50 copies/ml)	1:2	0.02	49	98	26.961	< 0.001	1 (reference)
	Low level viraemia (51–999 copies/ml)							18.429 (4.989–68.071)
	Virological failure (> 1000 copies/ml)							22.567 (6.958–73.191)
Treatment interruption ^c^	Treatment interruption (No)	1:1	0.01	55	55	13.274	0.001	1 (reference)
	Treatment interruption (Yes)							14.000 (3.335–58.768)
Treatment interruption ^c^	Treatment interruption (No)	1:1	0.02	57	57	11.814	< 0.001	1 (reference)
	Treatment interruption (Yes)							12.500 (2.961–52.773)
Treatment interruption ^c^	Treatment interruption (No)	1:2	0.01	46	92	17.377	< 0.001	1 (reference)
	Treatment interruption (Yes)							13.110 (3.910–43.963)
Treatment interruption ^c^	Treatment interruption (No)	1:2	0.02	49	98	20.501	< 0.001	1 (reference)
	Treatment interruption (Yes)							9.261 (3.534–24.273)

^a^Adjusted for age (≥ 60 years vs < 60 years), body mass index (< 18.5, 18.5–24.9, > 25.0), family members (< 3 individuals vs ≥ 3 individuals), gender, residence, region, occupation, nationality, marital status, educational level, toilet type, water supply, drinking water, livestock, pet, HIV infection route, newly infection, HIV VL, treatment interruption

^b^Adjusted for age (≥ 60 years vs < 60 years), body mass index (< 18.5, 18.5–24.9, ≥ 25.0 kg/m^2^), family members (< 3 individuals vs ≥ 3 individuals), gender, residence, region, occupation, nationality, marital status, educational level, toilet type, water supply, drinking water, livestock, pet, HIV infection route, newly infection, CD4^+^ T cell counts, duration of treatment interruption in HARRT. c: adjusted for age (≥ 60 years vs < 60 years), body mass index (< 18.5, 18.5–24.9, ≥ 25.0 kg/m^2^), family members (< 3 individuals vs ≥ 3 individuals), gender, residence, region, occupation, nationality, marital status, educational level, toilet type, water supply, drinking water, livestock, pet, HIV infection route, newly infection, CD4^+^ T cell counts, HIV VL

*CI*s: Confidence intervals. HAART: Highly active antiretroviral therapy. HIV: Human immunodeficiency virus.* OR*: Odds ratio. VL: Viral load

Similarly, under different matching ratios and caliper conditions, after adjusting for multiple confounding factors, the risk of Bh infection was found to have statistically significant differences among different levels of HIV virological status (full virological suppression, low level viraemia, virological failure) (*P* < 0.05). Specifically, the Bh infection in PLWH with virological failure was 10.784 to 147.072 times higher compared to the full virological suppression PLWH, while the risk of Bh infection in the low level viraemia group was 7.895 to 84.668 times higher compared to the full virological suppression PLWH (Table 2. Additional file 1: Tables S5, S6).

Finally, after applying different caliper levels (0.01, 0.02, 0.10, 0.20) and matching ratios (1:1, 1:2, 1:4), and adjusting for other confounding factors, the risk of Bh infection remained consistently higher in the PLWH with treatment interruption compared to the HIV/AIDS individuals who consistently adhered to antiviral treatment, ranging from 6.264 to 84.668 times higher (*P* < 0.001) in various scenarios (Table 2. Additional file 1: Table S5, S6).

Furthermore, when CD4^+^ T cell counts, HIV VL, and duration of interruption in HARRT were considered as continuous variables, after adjusting for confounding factors, the risk of Bh infection decreased with increasing CD4^+^ T cell counts (all *P* < 0.05), increased with rising HIV VL (all *P* < 0.05), and increased with longer duration of interruption in HARRT(all *P* < 0.05. Additional file 1: Tables S5, S6).

### RCS explore impact factors of Bh infection

A nonlinear relationship was observed between the risk of Bh infection and CD4^+^ T cell counts by modeling a RCS logistic regressions with three knots. The likelihood ratio test indicated that the nonlinear model was considerably better compared to the linear model (*χ*^2^ = 16.955, *P* < 0.001. Additional file 1: Table S7). Similarly, when exploring the association between the risk of Bh infection and HIV VL, logistic regression model based on RCS outperformed the standalone logistic regression model (*χ*^2^ = 30.242, *P* < 0.001. Additional file 1: Table S7). Additionally, when analyzing the association between the risk of Bh infection and duration of interruption in HARRT, the logistic regression model based on RCS demonstrated superior performance compared to the standalone logistic regression model (*χ*^2^ = 16.925, *P* < 0.001. Additional file 1: Table S7. Fig. S1).

**Fig. 2:**
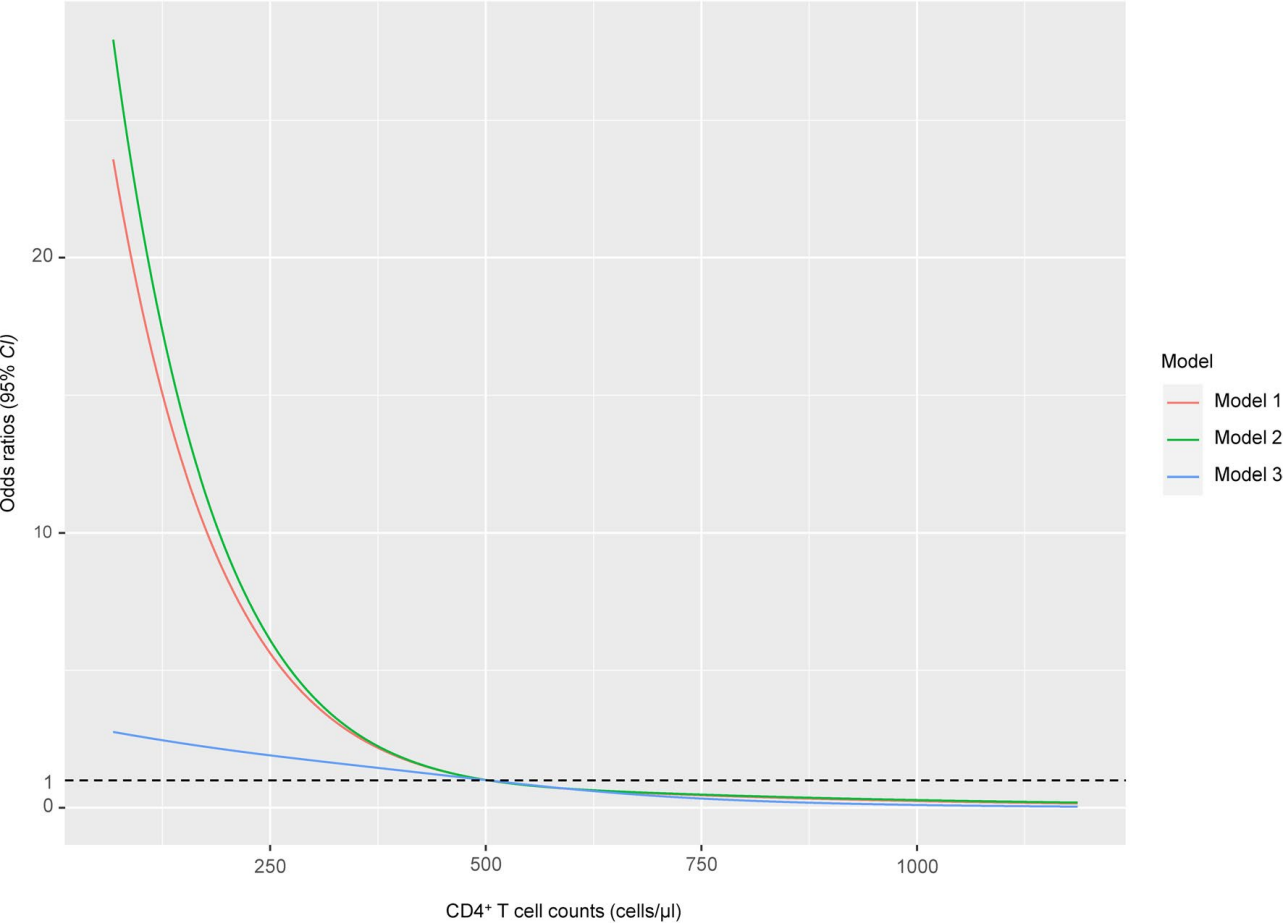
After adjustments in restricted cubic spline logistic multiple models, the sharp between *Blastocystis hominis* infection risk and CD4^+^ T cell counts. Model 1 only included CD4^+^ T cell counts. Model 2 was adjusted for gender, age, body mass index, nationality, residence, region, occupation, marital status, education level, family numbers, toilet type, water supply, drinking water, livestock, pet. Model 3 further adjusted for HIV infection route, HIV viral load, HIV infection time, and duration of interruption in highly active antiretroviral therapy

Nonlinear models revealed a significant L-shaped association between the Bh infection risk and CD4^+^ T cell counts. Despite variations in the *OR* value at lower CD4^+^ T cell counts was low among different models, all three models consistently exhibited the L-shaped relationship (all *P*_non-linearity_ < 0.001). Notably, the risk of Bh infection significantly decreased within the CD4^+^ T cell rose from 0 cells/μl to 500 cells/μl. However, once CD4^+^ T cell counts exceeded 500 cells/μl, the risk of Bh infection remained at a low and stable level as CD4^+^ T cell counts increased, the value of the range falls between 0.0 and 1.0, and it gradually approaches 0.0 as CD4^+^ T cell counts increases (Fig. 2).

**Fig. 3:**
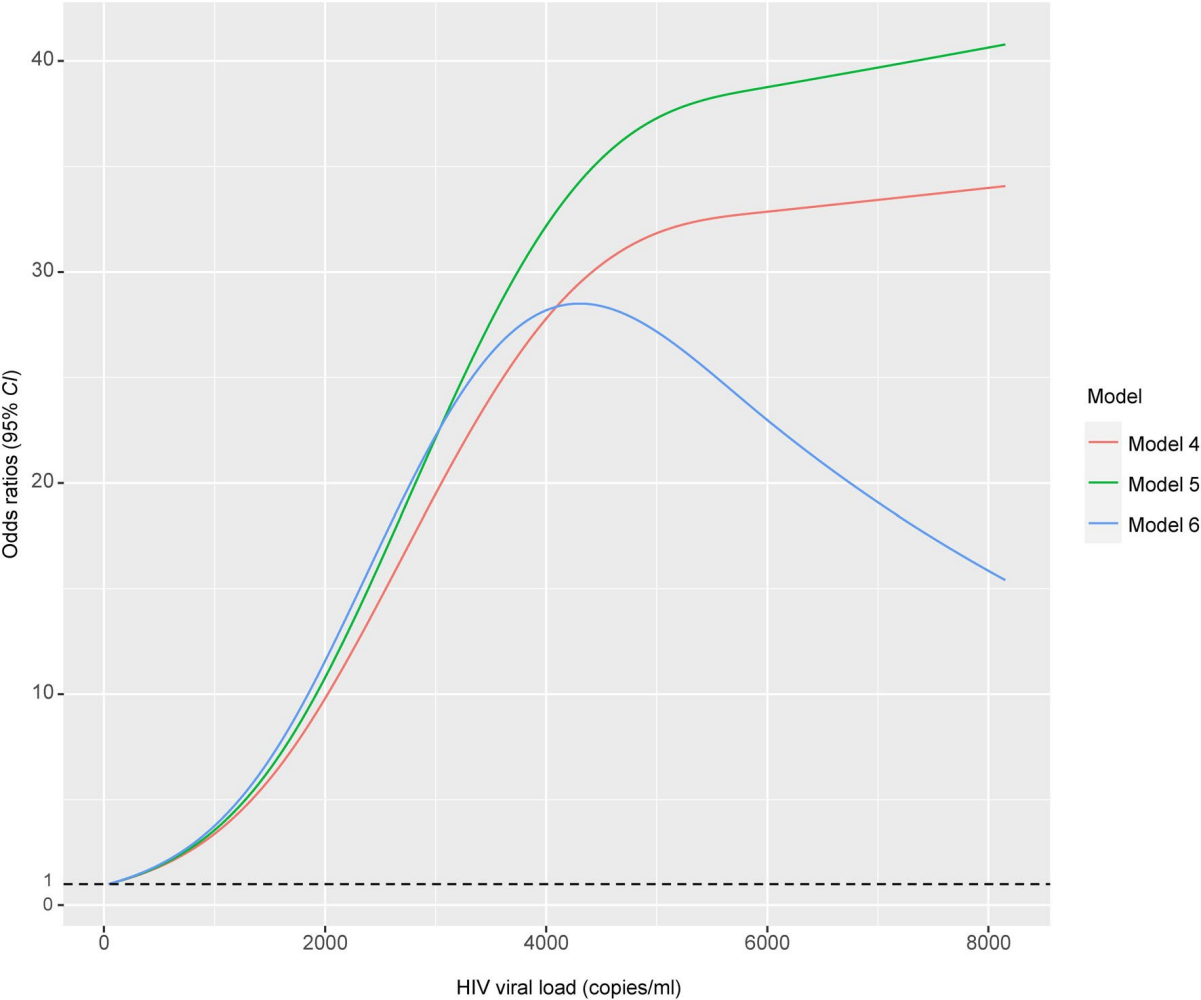
After adjustments in restricted cubic spline logistic multiple models, the sharp between *Blastocystis hominis* infection risk and HIV viral load. Model 4 only included HIV viral load. Model 5 was adjusted for gender, age, body mass index, nationality, residence, region, occupation, marital status, education level, family numbers, toilet type, water supply, drinking water, livestock, pet. Model 6 further adjusted for HIV infection route, CD4^+^ T cell counts, HIV infection time, and duration of interruption in highly active antiretroviral therapy

There is a significant nonlinear association (all *P*_non-linearity_ < 0.001) between the Bh infection risk and HIV VL, these curves manifest different trends depending on the adjusted factors. In model 4 and 5, the relationship between the risk of Bh infection and HIV VL exhibits a distinct S-shaped pattern. It implies that as HIV VL increases, the risk of Bh infection rapidly rises until reaching a plateau when HIV VL reaches a range of 4000 to 5000 (copies/ml), and then maintaining a higher level (*OR *> 35.0). However, after adjusting for all measured confounding factors in model 6, the association between the risk of Bh infection and HIV VL displays an inverted U-shaped pattern. The highest odds ratio (*OR* = 28.499, 95% *CI*: 8.430–96.345) is observed at a HIV VL of 4320 copies/ml. Prior to reaching 4320 copies/ml, the risk of Bh infection increases rapidly with increasing HIV VL. However, after reaching 4320 copies/ml, the risk of Bh infection gradually decreases with further increases in VL, albeit remaining at a relatively high level (*OR* > 15.0. Fig. 3).

**Fig. 4:**
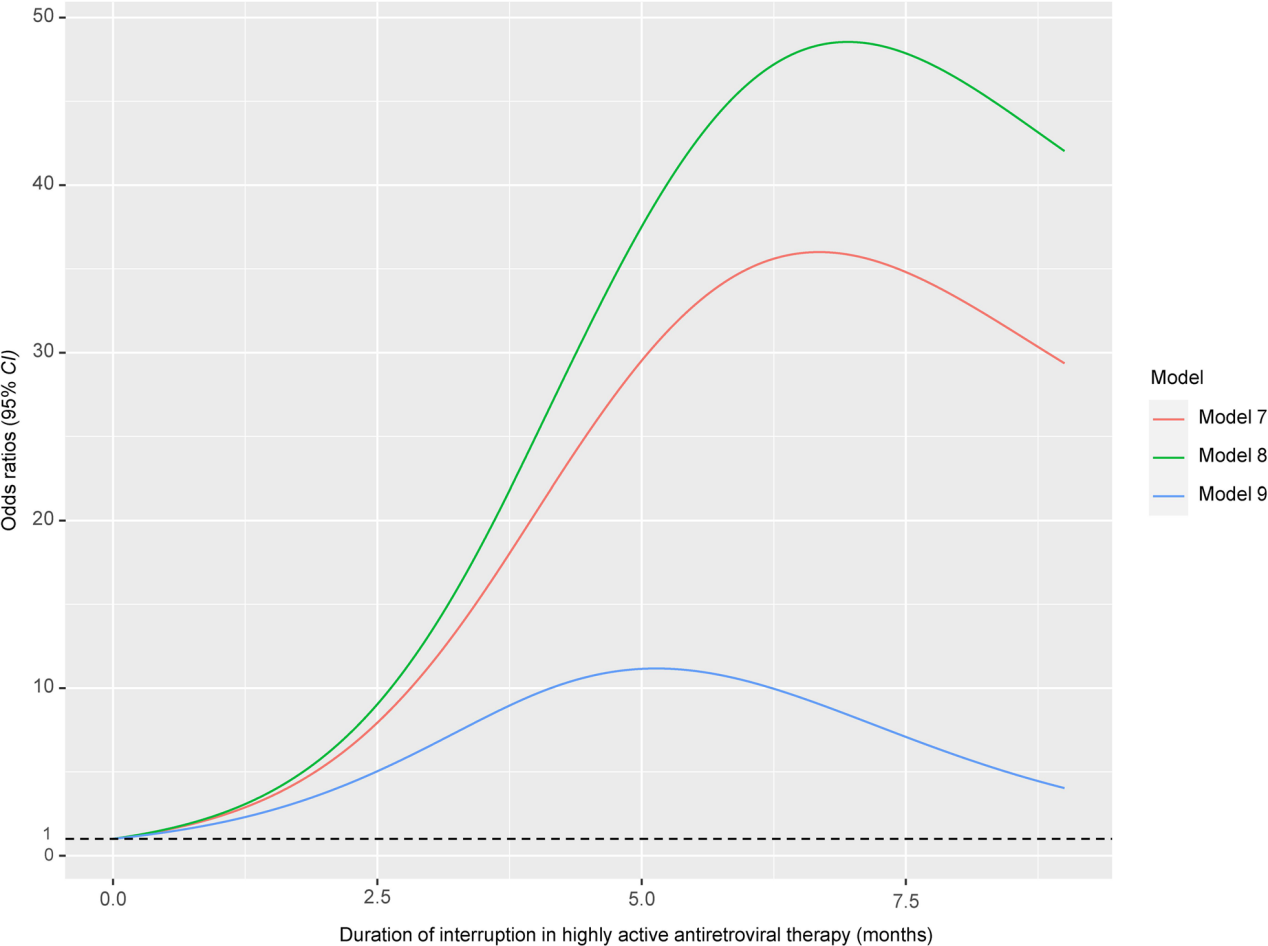
After adjustments in restricted cubic spline logistic multiple models, the sharp between *Blastocystis hominis* infection risk and duration of interruption in highly active antiretroviral therapy. Model 7 only included duration of interruption in highly active antiretroviral therapy. Model 8 was adjusted for gender, age, body mass index, nationality, residence, region, occupation, marital status, education level, family numbers, toilet type, water supply, drinking water, livestock, pet. Model 9 further adjusted for HIV infection route, CD4^+^ T cell counts, HIV viral load, and HIV infection time

Nonlinear models revealed a significant inverted U-shaped association (all *P*_non-linearity_ < 0.001) between the risk of Bh infection and duration of interruption in HARRT. As confounding factors increased, overall, all risk of Bh infection remained consistently above 8.0. After adjusting for all confounding factors, Model 9 demonstrated a rapid increase in the risk of Bh infection following antiretroviral treatment interruption for more than 2 months, reaching its peak in the fifth month (*OR* = 11.152, 95%* CI*: 4.291, 28.378). Subsequently, from the fifth to the twelfth month, the risk of Bh infection gradually decreased but still maintained a relatively high level (Fig. 4).

### Sensitivity analysis results

E-value analyses was conducted to find the robustness of associations between Bh infection and CD4^+^ T immunological status, HIV virological status and treatment interruption. Moderately robust of unmeasured confounding factors was observed (Additional file 1: Table S8). For example, the minimum value required for the joint minimum strength of association of the risk ratio scale for an unmeasured confounder with the mild immunosuppression and no immunosuppression groups to fully explain an observed treatment-outcome risk ratio of relative risk (*RR*) was 1.796 in PLWH. The E-value for this was 17.293, it means that residual confounding could explain the observed association if there exists an unmeasured covariate having a relative risk association at least as large as 17.293 between Bh infection and moderate immunosuppression, compared of no immunosuppression of PLWH.

Compared with full virological suppression, an associated with Bh infection and low-level viraemia by risk ratios of 4.837, each above and beyond the large array of potential confounder already adjusted for, could explain away the association, but weaker confounding could not, to shift the confidence interval to include the null. Further, based on the calculated E-values, an unmeasured confounder would need to be associated with both the risk of Bh infection and virological failure by a magnitude of 10.221-fold above and beyond the measured variable included in the model to explain away the observed associations. In addition, E-value calculations showed that an unmeasured confounder would need to have nearly 1.437 times as large an effect as treatment interruption, and be associated with both the exposure and the outcome to completely explain away the observed associations.

The lasso logistic regression method was utilized to identify the most significant predictive factors for Bh infection in HIV/AIDS individuals. Feature selection was performed using the complete database (incorporating a total of 19 features). Finally, three features with nonzero coefficients were ultimately selected, including CD4^+^ T immunological status, HIV virological status, and treatment interruption (Additional file 1: Table S9). Furthermore, feature selection considering age, BMI, HIV infection time, CD4^+^ T cell counts, HIV VL, and duration of interruption in HARRT as continuous variables. The final results revealed that CD4^+^ T cell counts and HIV VL were influential factors in Bh infection among PLWH (Additional file 1: Table S9).

Four algorithms (RF, ANN, GBM, and XGBOOST) were employed to determine the important variables that influencing Bh infection in PLWH. All model constructions were based on the training datasets. The dependent variable was Bh infection, and all independent variables (including 19 variables such as CD4^+^ T immunological status, HIV virological status, and treatment interruption) were included in the analysis as categorical variables. The RF model, ANN model, GBM model, and XGBOOST model had an AUROC of 0.887 (95% *CI*: 0.810–0.964), 0.824 (95% *CI*: 0.741–0.906), 0.882 (95% *CI*: 0.830–0.934), and 0.874 (95% *CI*: 0.818–0.930), respectively (Additional file 1: Fig. S2). Among four models, the accuracy ranged from 80.6% to 84.8%, while the sensitivity ranged from 90.5% to 97.6%, the specificity ranged from 79.7% to 84.5%, and the F1 score values ranged from 0.83 to 0.88. These metrics demonstrate the performance of each model, and all four models exhibit good performance, effectively extracting the relevant features that influence Bh infection (Additional file 1: Table S10). In addition, four models identified the top three variables with predictive value for Bh infection as CD4^+^ T immunological status, HIV virological status, and treatment interruption (Additional file 1: Fig. S3).

Furthermore, when incorporating seven continuous variables (age, BMI, HIV infection time, CD4^+^ T cell counts, HIV VL, and duration of interruption in HARRT) and other categorical variables into these four machine learning models (Additional file 1: Fig. S4), the results found that four models had AUROC values above 0.750 (all *P* < 0.05). The accuracy ranged from 81.7% to 86.6%, while the sensitivity ranged from 89.7% to 95.1%, the specificity ranged from 81.4% to 86.4%, and the F1 score values ranged from 0.83 to 0.85. The evaluation metrics of each model indicate that they perform well in terms of predictive performance. The models accurately reflect the factors that influence Bh infection (Additional file 1: Table S10). Moreover, these four models consistently identified HIV VL, CD4^+^ T cell counts, and duration of interruption in HARRT as the top three influencing factors for Bh infection (Additional file 1: Fig. S5).

## Discussion

Through a large-scale and multi-center observational study, it has identified that CD4^+^ T cell counts and HIV VL as crucial markers for the risk of Bh infection among PLWH. Notably, the study accurately revealed dynamic changes and quantitative non-linear association between the risk of Bh infection and CD4^+^ T cell counts, as well as HIV VL, using RCS models. These results significantly contribute to the understanding of HIV/AIDS disease progression, early identification of Bh infection, optimization of treatment approaches, and evaluation of treatment outcomes for PLWH. The findings provide valuable insights for healthcare professionals and policymakers, guiding them to implement timely and comprehensive intervention measures for PLWH to reduce the incidence of both Bh and other opportunistic pathogens, ultimately contributing to a decrease in HIV-related mortality.

### Bh infection risk and CD4^+^ T cell counts

Based on the positive collaborative history from previous projects and the willingness of physicians from sentinel hospitals to participate in the study, these three cities were selected from the multitude of options as study sites. The study found that the risk of Bh infection decreases rapidly with increasing CD4^+^ T cell counts, and after reaching 500 cells/μl, Bh infection risk remains consistently low level. CD4^+^ T cell counts reflects the degree of immune deficiency among PLWH, a natural decline in CD4^+^ T cell counts is one of the gold standards for assessing disease progression in PLWH. Additionally, CD4^+^ T cell counts can be used to monitor the OPIs occurrence, as there is a strong correlation between CD4^+^ T cell counts and the OPIs emergence. Numerous studies shown that when CD4^+^ T cell counts fall below 200 cells/μl, many OPIs (Bh, *Cryptosporidium* spp., *Giardia lamblia*, *Toxoplasma gondii*, *Klebsiella* spp*.*, etc.) will emergence [42, 43], and PLWH do not seek medical attention at hospitals or clinics when they experience symptoms related to OPIs. Instead, they self-medicate or even neglect seeking any treatment, it may exacerbate their condition and increase mortality. Our study showed that although the Bh infection risk decreases as CD4^+^ T cell counts range from 200 cells/μl to 500 cells/μl, the absolute risk remains significant. With the emergence of new effective antiviral drugs, controlling viral replication in PLWH is no longer a challenge, and clinical attention has shifted to the restoration of immune function after HAART. Therefore, CD4^+^ T cell counts can serve as a biological indicator for both Bh and other opportunistic pathogens in PLWH. When CD4^+^ T cell counts drop below 500 cells/μl (especially below 200 cells/μl), additional testing and monitoring of common opportunistic pathogens are necessary to achieve early intervention and treatment for HIV-related OPIs, and improving the quality of life for PLWH.

The study found that when CD4^+^ T cell counts exceeded 500 cells/μl, the Bh infection consistently remained at lower levels, which aligns with our experience and understanding. Additionally, as the number of covariates increased, the presence of other important variables influenced the risk of Bh infection in conjunction with CD4^+^ T cell counts within the range of 0 to 500 cells/μl. In other words, compared to CD4^+^ T cells, other variables such as HIV VL exerted a greater impact on Bh infection.

### Bh infection risk and HIV VL

The study investigated the association between the risk of Bh infection and HIV VL using non-linear model, it revealed that HIV VL was the most robust independent indicator associated with Bh infection risk. As the HIV VL increased, the prevalence of Bh also rose when the HIV VL was below 4320 copies/ml. The study demonstrated that increasing VL was independently associated with increased risk, it was consistent with other study [44]. Furthermore, the study corroborates previous conclusions that HIV VL shows an inverse correlation with CD4^+^ T cell counts [45]. HIV infection invades the lymphocytes of the host, leading to compromised immune function and progressive depletion and functional limitations of CD4^+^ T cells. This immune impairment contributes to the various OPIs occurrence, including bacterial, viral, and parasitic infections, as well as opportunistic tumors, which are significant factors contributing to the mortality of PLWH. HIV VL serves as a direct indicator of antiviral therapy effectiveness and the infectivity of PLWH as potential transmission source. Compared to clinical and immune markers, it is a faster, more sensitive, stable, and robust biological marker, making it the optimal metric for evaluating treatment outcomes and assessing the transmission risk of PLWH.

It demonstrated that routine CD4^+^ T cell counts monitoring is more cost-effective than adding HIV VL testing to the monitoring strategy [46]. However, HIV VL testing is an essential component of antiretroviral treatment monitoring programs, as it helps assess treatment response and evaluate the need for treatment changes [47]. Furthermore, the study revealed that the decrease in HIV VL and increase in CD4^+^ T cell counts do not always align perfectly. Some PLWH with CD4^+^ T cell counts exceeding 500 cells/μl and their HIV VL also surpassing 1000 copies/ml. It suggests that although their immune function may not be significantly impaired, these individuals still exhibit active viral replication and high infectivity. The study suggested that HIV VL monitoring is particularly indicative for preventing both Bh and other opportunistic pathogens, considering the growing accessibility and affordability of fluorescence quantitative detection methods and diagnostic reagents, a combined approach of monitoring HIV VL and peripheral blood CD4^+^ T cell counts should be considered to conduct in some regions with relatively high levels of socioeconomic development. It will provide a better assessment of the OPIs risk, HIV/AIDS disease progression, the effectiveness of antiviral drug, and the outcomes of prevention and control measures.

The study found that the risk of Bh infection reaches its peak with increasing HIV VL, but contrary to our hypothesis, the risk of Bh infection does not gradually decrease as VL continues to increase. This finding may be attributed to the low detection rate of Bh (less than 5%) in the study. When the detection rate of Bh is low and the PLWH with high VL is limited, observational study results are prone to bias. Additionally, when HIV VL are high, it can be inferred from a certain perspective that PLWH experience treatment interruption, have longer disease duration, exhibit poor adherence, are reluctant to participate in such studies, or have died. These factors contribute to significant biases to a certain extent.

### Bh infection risk and duration of treatment interruption in HARRT

The most notable finding of our study is the gradual increase in the risk Bh infection with an extended duration of interruption in HARRT, reaching its peak and subsequently declining slowly while remaining at a relatively higher risk level. This highlights the significant impact of continuous and standardized HARRT to keep health for PLWH. Promoting HAART was an essential method to achieve the first 90% of the global “90–90-90” target (90% detection, 90% treatment, 90% viral suppression) by 2020, and the second 95% of the upgraded global “95–95-95–95” target (95% combination prevention, 95% detection, 95% treatment, 95% viral suppression) [9]. HAART adherence is considered a crucial predictor for virological suppression. Treatment interruption can lead to a rapid increase of HIV VL, consequently compromising the functionality of immune cells (including CD4^+^ T cell counts), and resulting in the OPIs occurrence, OPIs continuing increases in PLWH between 4 and 8 years after starting HAART, regardless of CD4^+^ T cell counts at initiation of antiviral therapy [48]. PLWH should be encouraged to initiate and maintain HARRT at the earliest opportunity. Healthcare professionals involved in HIV diagnosis and treatment should routinely monitor patients' CD4^+^ T cell counts, HIV VL, drug resistance profiles, and other biological markers. Building upon standardized treatment protocols, personalized therapeutic strategies should be developed to cater to the unique needs of each patient.

It is imperative to reinforce HIV VL testing and implement educational campaigns to raise awareness about antiviral therapy, thereby increasing the proportion of PLWH receiving optimal treatment. Medication adherence plays a pivotal role in the antiviral therapy success. Thus, adherence education should be prioritized prior to initiating HAART in all PLWH. In addition, high prevalence of HIV drug resistance would restrict therapy options, compromise the effect of current therapy regimens, and increase the risk of treatment failure [9]. A crucial aspect is the continuous monitoring of the interrelationship between the type, dosage, and VL of antiviral medications during the long-term treatment follow-up process. The monitoring aids for the ongoing optimization of the HAART regimen, thereby mitigating the risk of treatment interruption. Upon confirmation of an HIV infection, irrespective of the CD4^+^ T lymphocyte levels, it is strongly recommended to promptly commence standardized and continuous antiviral therapy. Such an approach effectively suppresses viral replication, resulting in a reduction of viral load to undetectable levels and minimizing viral mutations. This subsequently facilitates immune function restoration, reduces the likelihood of abnormal immune activation, curbs viral transmission, lowers HIV-related mortality rates, decreases the incidence and mortality rates of non-AIDS-related diseases, ultimately enabling PLWH to attain a normal life expectancy and enhance their overall quality of life.

The interactions between HIV and opportunistic pathogenic bacteria, as well as other intestinal parasites, are well-documented and significantly impact the health status of individuals with HIV/AIDS. HIV-positive individuals often exhibit a high prevalence of opportunistic intestinal pathogens, and chronic AIDS patients with low CD4^+^ T cell counts frequently demonstrate polyparasitic infections [42]. The substantial presence of HIV is responsible for the deterioration of immune cells, especially the target CD4^+^ T cells, thus aiding the entry of OPIs that exacerbating HIV progression or increasing HIV transmission. Episodes of OPIs among patients with undetectable VL were associated with elevation of HIV VL to detectable levels and the decline in CD4^+^ T cell counts [49]. Hence, it was evident that the association between OPIs, co-infections, and HIV infection is characterized by a reciprocal and mutually reinforcing interaction [50].

The found that the risk of Bh infection reaches its peak after a certain duration of treatment interruption and subsequently shows a slight decline. This finding is inconsistent with our hypothesis, which suggests that the risk of Bh infection continuously increases with the duration of treatment interruption. This discrepancy may be related to the relatively small proportion of PLWH experiencing treatment interruption. When this subset of PLWH is limited, significant biases can occur in the occurrence of Bh infection. Therefore, future studies with expanded sample sizes will provide more accurate insights into the curve association between the Bh infection risk and treatment interruption.

Although the study provides valuable evidence and insights into Bh infection among PLWH, there are several limitations that need to be acknowledged. Firstly, there may have been selection bias due to its cross-sectional design, it may lead to significant differences of baseline characteristics between Bh positive cases and Bh negative individuals. Secondly, other biometric markers (CD8^+^ T cells counts, CD4^+^ T/CD8^+^ T, etc.) were not measured, limiting the comprehensive understanding of factors contributing to Bh and opportunistic pathogens infection among PLWH. Thirdly, the study was unable to establish the temporal sequence for anti-HIV drug interruption time, CD4^+^ T cell counts and HIV VL, thus the study cannot determine the causal association among these variables. Fourthly, other intestinal pathogens were not detection [51–53], and co-infections of enteric pathogens were not considered in the study [51, 52]. Therefore, future study should focus on large-scale, multi-center prospective observational study to more accurately elucidate the association among immune status, antiviral treatment and the OPIs in PLWH.

## Conclusions

The study demonstrated a significant association between an elevated risk of Bh infection and lower CD4^+^ T cell counts, higher HIV VL, and longer treatment interruption durations among PLWH. Furthermore, significant nonlinear association were observed between Bh infection risk and CD4^+^ T cell counts, HIV VL, as well as duration of treatment interruption. In addition, the significance of HIV VL was greater than CD4^+^ T cell counts in aspects of Bh emergence. The combined surveillance of HIV VL and peripheral blood CD4^+^ T counts is recommended in the regions with a high level of socioeconomic development, as it provides a more comprehensive and surveillance assessment of Bh infection and other OPIs, disease progression, treatment efficacy of antiviral drug, and the effectiveness of preventive measures. Additionally, it is crucial to employ various effective strategies and management methods to reduce loss to follow-up during the implementation of antiretroviral therapy, promote continuous treatment adherence, enhance the immune cell count, suppress VL, decrease OPIs, and improve the quality of life for PLWH.

### Supplementary Information

**Additional file 1: Table S1.** Testing of normality for age, body mass index, HIV infection time, duration of interruption in highly active antiretroviral therapy, CD4^+^ T cell counts, HIV viral load. **Table S2.** Comparison of some quantitative variables in persons living with human immunodeficiency virus with or without *Blastocystis hominis* infection. **Table S3.** The correlation between CD4^+^ T cell counts, HIV viral load, duration of HIV infection, the duration of interruption in highly active antiretroviral therapy in people living with HIV. **Table S4.** Multivariable analysis with logistic regression for *Blastocystis hominis* infection in persons living with human immunodeficiency virus. **Table S5.** Propensity score method was used to explore the association between *Blastocystis hominis* infection and CD4^+^ T immunological status, HIV virological status, and treatment interruption. **Table S6.** The study employs propensity scores to investigate the association between *Blastocystis hominis* infection and CD4^+^ T cell counts (cells/μl), HIV viral load (copies/ml), and the duration of interruption in antiretroviral therapy (month). **Table S7**. Univariate linear models and nonlinear models was used to investigate the relationship between *Blastocystis hominis* infection and CD4^+^ T cell counts (cells/μl), HIV viral load (copies/ml), and the duration of interruption in highly active antiretroviral therapy (month). **Table S8.** E-value analyses were employed to examine the strength and robustness of associations between *Blastocystis hominis* infection and CD4+ T immunological status, HIV virological status, as well as treatment interruption. **Table S9.** The least absolute shrinkage and selection operator (lasso) logistic regression was conducted to explore the risk factors associated with *Blastocystis hominis* infection in individuals living with human immunodeficiency virus/acquired immune deficiency syndrome. **Table S10.** Model evaluation indicators for these four algorithms. **Fig. S1.** Restricted cubic spline (RCS, three knots, 10th, 50th and 90th percentiles) was applied to explore the association between *Blastocystis hominis* infection risk and HIV infection time (year), CD4^+^ T counts, HIV viral load and duration of interruption in highly active antiretroviral therapy (month). **Fig. S2.** The four algorithms were applied for screening of risk factors associated with *Blastocystis hominis* infection in persons living with HIV (area under curve. categorical variable). **Fig. S3.** The four algorithms were adopted for screening of the risk factors associated with *Blastocystis hominis* infection in person living with HIV (feature selection. Categorical variable). **Fig. S4.** The four algorithms were applied for demonstrating the all risk factors associated with *Blastocystis hominis infection* in person living with HIV (area under curve. Continuous variable). **Fig. S5.** The four algorithms were adopted for indicating the all risk factors associated with *Blastocystis hominis* infection in persons living with HIV (feature selection. Continuous variable).

## Data Availability

The datasets generated during and/or analyzed in this study are available from the first author on reasonable request (Email: zhangshunxian110@163.com).

## Abbreviations

AI: Artificial intelligence
AIDS: Acquired Immune Deficiency Syndrome
ANN: Artificial neural network
ART: Antiviral therapy
AUROC: Area under the receiver operating characteristic curve
Bh: *Blastocystis hominis*
BMI: Body mass index
CD4^+^ T: CD4^+^ T-lymphocyte
CDC: Center for Disease Control and Prevention
*CI*s: Confidence intervals
COVID-19: Coronavirus disease 2019
CRIMS: Comprehensive Response Information Management System
*Ct*: Cycle threshold
DNA: Deoxyribonucleic acid
EDTA-k2: Ethylenediaminetetraacetic acid dipotassium salt dihydrate
GBM: Gradient boosting machine
HAART: Highly active antiretroviral therapy
HIV: Human immunodeficiency virus
VL: Viral load
IQR: Inter-quartile range
KS: Kolmogorov–Smirnov
Lasso: Least absolute shrinkage and selection operator
MI: Multiple imputation
NFAPT: National Free Antiretroviral Treatment Program
OPIs: Opportunistic infections
*OR*: Odds ratio
PBMCs: Peripheral blood mononuclear cells
PBS: Phosphate buffered saline
PLWH: Persons living with human immunodeficiency virus
PS: Propensity score
PSM: Propensity score matching
qPCR: Quantitative polymerase chain reaction
RCS: Restricted cubic spline
RNA: Ribonucleic acid
RF: Random forest
*RR*: Relative risk
STROBE: Strengthening the Reporting of Observational Studies in Epidemiology
UNAIDS: The Joint United Nations Programme on HIV/AIDS
XGBOOST: EXtreme gradient boosting
