# Supplementary material for: Impact factors of Blastocystis hominis infection in persons living with human immunodeficiency virus: a large-scale, multi-center observational study from China
Source: Infect Dis Poverty. 2023 Sep 11;12:82. doi: 10.1186/s40249-023-01137-5 (PMC10494452; doi:10.1186/s40249-023-01137-5)
Supplement: Supplementary file 1 — Additional file 1: Table S1. Testing of normality for age, body mass index, HIV infection time, duration of interruption in highly active antiretroviral therapy, CD4+ T cell counts, HIV viral load. Table S2. Comparison of some quantitative variables in persons living with human immunodeficiency virus with or without Blastocystis hominis infection. Table S3. The correlation between CD4+ T cell counts, HIV viral load, duration of HIV infection, the duration of interruption in highly active antiretroviral therapy in people living with HIV. Table S4. Multivariable analysis with logistic regression for Blastocystis hominis infection in persons living with human immunodeficiency virus. Table S5. Propensity score method was used to explore the association between Blastocystis hominis infection and CD4+ T immunological status, HIV virological status, and treatment interruption. Table S6. The study employs propensity scores to investigate the association between Blastocystis hominis infection and CD4+ T cell counts (cells/μl), HIV viral load (copies/ml), and the duration of interruption in antiretroviral therapy (month). Table S7. Univariate linear models and nonlinear models was used to investigate the relationship between Blastocystis hominis infection and CD4+ T cell counts (cells/μl), HIV viral load (copies/ml), and the duration of interruption in highly active antiretroviral therapy (month). Table S8. E-value analyses were employed to examine the strength and robustness of associations between Blastocystis hominis infection and CD4+ T immunological status, HIV virological status, as well as treatment interruption. Table S9. The least absolute shrinkage and selection operator (lasso) logistic regression was conducted to explore the risk factors associated with Blastocystis hominis infection in individuals living with human immunodeficiency virus/acquired immune deficiency syndrome. Table S10. Model evaluation indicators for these four algorithms. Fig. S1. Restricted cubi [file 40249_2023_1137_MOESM1_ESM.docx]

**Additional file 1**

**The impact factors of *Blastocystis hominis* infection in persons living with human immunodeficiency virus: a large-scale, multi-center observational study from China**

Shun-Xian Zhang^1,2,3#，^Ji-Chun Wang^4#^, Zhong-Wei Li^5#^, Jin-Xin Zheng^2,3^, Wen-Ting Zhou^6^, Guo-Bing Yang^7^, Ying-Fang Yu^2,3^, Xiu-Ping Wu^2,3^, Shan Lv^2,3^, Qin Liu^2,3^, Mu-Xin Chen^2,3^, Yan Lu^2,3^, Zhi-Hui Dou^8^, Da-Wei Zhang^9^, Wen-Wen Lv^10^, Lei Wang^1^, Zhen-Hui Lu^1^, Ming Yang^1^, Pei-Yong Zheng^1^, Yue-Lai Chen^1^, Li-Guang Tian^2,3*^, Xiao-Nong Zhou^2,3*^

^1^ Longhua Hospital, Shanghai University of Traditional Chinese Medicine, Shanghai 200032, China.

^2^ Chinese Center for Disease Control and Prevention (Chinese Center for Tropical Diseases Research), NHC Key Laboratory of Parasite and Vector Biology, WHO Collaborating Centre for Tropical Diseases, National Center for International Research On Tropical Diseases, National Institute of Parasitic Diseases, Shanghai 200025, China.

^3^ School of Global Health, Chinese Center for Tropical Diseases Research-Shanghai Jiao Tong University School of Medicine, Shanghai, 200025 China.

^4^ Department of Science and Technology, Chinese Center for Disease Control and Prevention, Beijing 102206 China.

^5^ Gansu Province People's Hospital, Gansu Provincial Hospital, Lanzhou 730000, China

^6^ National Health Commission (NHC) Key Laboratory of Biosafety, National Institute for Viral Disease Control and Prevention, Chinese Center for Disease Control and Prevention, Beijing 102206, China.

^7^ Gansu Provincial Center For Disease Control and Prevention, Lanzhou 730000, China.

^8^ National Center for AIDS/STD Control and Prevention, China Center for Disease Control and Prevention, Beijing 102206, China.

^9^ The People's Liberation Army 302 Hospital, Beijing 100039, China.

^10^ Clinical Research Institute, Shanghai Jiao Tong University School of Medicine, Shanghai, 200025, China.

^#^ Shun-Xian Zhang, Ji-Chun Wang, Zhong-Wei Li contributed equally to this work.

* Correspondence: Li-Guang Tian, [jztlg@126.com](mailto:jztlg@126.com);

Xiao-Nong Zhou, [zhouxn1@chinacdc.cn](mailto:zhouxn1@chinacdc.cn).

**Supplementary Tables**

**Table S1.** Testing of normality for age, body mass index, HIV infection time, duration of interruption in highly active antiretroviral therapy, CD4^+^ T cell counts, HIV viral load.

**Table S2.** Comparison of some quantitative variables in persons living with human immunodeficiency virus with or without *Blastocystis hominis* infection.

**Table S3.** The correlation between CD4^+^ T cell counts, HIV viral load, duration of HIV infection, the duration of interruption in highly active antiretroviral therapy in people living with HIV.

**Table S4.** Multivariable analysis with logistic regression for *Blastocystis hominis i*nfection in persons living with human immunodeficiency virus.

**Table S5.** Propensity score method was used to explore the association between *Blastocystis hominis* infection and CD4^+^ T immunological status, HIV virological status, and treatment interruption.

**Table S6.** The study employs propensity scores to investigate the association between *Blastocystis hominis* infection and CD4^+^ T cell counts (cells/μl), HIV viral load (copies/ml), and the duration of interruption in antiretroviral therapy (month).

**Table S7**. Univariate linear models and nonlinear models was used to investigate the relationship between *Blastocystis hominis* infection and CD4^+^ T cell counts (cells/μl), HIV viral load (copies/ml), and the duration of interruption in highly active antiretroviral therapy (month). **Table S8.** E-value analyses were employed to examine the strength and robustness of associations between Blastocystis hominis infection and CD4+ T immunological status, HIV virological status, as well as treatment interruption.

**Table S9.** The least absolute shrinkage and selection operator (lasso) logistic regression was conducted to explore the risk factors associated with *Blastocystis hominis* infection in individuals living with human immunodeficiency virus/Acquired Immune Deficiency Syndrome. **Table S10.**  Model evaluation indicators for these four algorithms.

**Supplementary Figures**

**Fig. S1.** Restricted cubic spline (RCS, three knots, 10^th^, 50^th^ and 90^th^ percentiles) was applied to explore the association between *Blastocystis hominis* infection risk and HIV infection time (year), CD4^+^ T counts, HIV viral load and duration of interruption in highly active antiretroviral therapy (month).

**Fig. S2.** The four algorithms were applied for screening of risk factors associated with *Blastocystis hominis* infection in persons living with HIV (area under curve. categorical variable).

**Fig. S3.** The four algorithms were adopted for screening of the risk factors associated with *Blastocystis hominis* infection in person living with HIV (feature selection. Categorical variable).

**Fig. S4.** The four algorithms were applied for demonstrating the all risk factors associated with *Blastocystis hominis infection* in person living with HIV (area under curve. Continuous variable).

**Fig. S5.** The four algorithms were adopted for indicating the all risk factors associated with *Blastocystis hominis* infection in persons living with HIV (feature selection. Continuous variable).

**Table S1.** Testing of normality for age, body mass index, HIV infection time, duration of interruption in highly active antiretroviral therapy, CD4^+^ T cell counts, HIV viral load.

| **Variables** | **Subgroups** | **Kolmogorov-Smirnov^a^** | | | **Shapiro-Wilk** | | |
| --- | --- | --- | --- | --- | --- | --- | --- |
|  |  | **Statistic** | **df** | ***P-value*** | **Statistic** | **df** | ***P-value*** |
| Age (Year) | Bh negative | 0.044 | 1,184 | <0.001 | 0.990 | 1,184 | <0.001 |
|  | Bh positive | 0.050 | 61 | 0.200* | 0.988 | 61 | 0.808 |
| BMI (kg/m^2^) | Bh negative | 0.054 | 1,184 | <0.001 | 0.982 | 1,184 | <0.001 |
|  | Bh positive | 0.074 | 61 | 0.200* | 0.982 | 61 | 0.489 |
| Family members (individuals) | Bh negative | 0.146 | 1,184 | <0.001 | 0.929 | 1,184 | <0.001 |
|  | Bh positive | 0.165 | 61 | <0.001 | 0.935 | 61 | 0.003 |
| HIV infection time (years) | Bh negative | 0.133 | 1,184 | <0.001 | 0.922 | 1,184 | <0.001 |
|  | Bh positive | 0.197 | 61 | <0.001 | 0.880 | 61 | <0.001 |
| Duration of interruption in highly active antiretroviral therapy (month) | Bh negative | 0.509 | 1,184 | <0.001 | 0.245 | 1,184 | <0.001 |
|  | Bh positive | 0.228 | 61 | <0.001 | 0.821 | 61 | <0.001 |
| CD4^+^ T cell counts (cells/μl) | Bh negative | 0.093 | 1,184 | <0.001 | 0.954 | 1,184 | <0.001 |
|  | Bh positive | 0.121 | 61 | 0.027 | 0.768 | 61 | <0.001 |
| HIV viral load (copies/ml) | Bh negative | 0.445 | 1,184 | <0.001 | 0.376 | 1,184 | <0.001 |
|  | Bh positive | 0.161 | 61 | <0.001 | 0.822 | 61 | <0.001 |

Notes: * It is a lower bound of the true significance. ^a^ Lilliefors Significance Correction. Bh: *Blastocystis hominis.* BMI:Body mass index*.* HIV: Human immunodeficiency virus.

**Table S2.** Comparison of some quantitative variables in persons living with human immunodeficiency virus with or without *Blastocystis hominis* infection.

| **Variables** | **Total**  **Median (*IQR*)** | **Bh**  **negative**  **Median (*IQR)*** | **Bh**  **positive**  **Median**  **(*IQR*)** | **Mann-**  **Whitney *U*** | **Wilcoxon**  ***W*** | ***Z*** | ***P-value*** |
| --- | --- | --- | --- | --- | --- | --- | --- |
| Age  (Year) | 43 (33, 52) | 43 (33, 52) | 45 (37, 52) | 33,191.5 | 734,711.5 | -1.067 | 0.286 |
| BMI (kg/m^2^) | 21 (20, 22) | 21 (20, 22) | 21 (20, 22) | 34,359 | 735,879 | -0.640 | 0.522 |
| Family members  (individuals) | 4 (2, 5) | 4 (2, 5) | 3 (3, 5) | 33,799.5 | 735,319.5 | -0.859 | 0.390 |
| HIV infection  Time (year) | 7 (3, 13) | 7 (3, 13) | 12 (4, 14) | 29,526.5 | 731,046.5 | -2.414 | 0.016 |
| Duration of interruption in highly active antiretroviral therapy (month) | 0 (0, 0) | 0 (0, 0) | 2 (0, 5) | 17,764.5 | 719,284.5 | -12.539 | <0.001 |
| CD4^+^ T cell count (cells/μl) | 502 (389, 603) | 511 (403, 610) | 250 (178, 386) | 11,943.5 | 138,34.5 | -8.826 | <0.001 |
| HIV viral load  (copies/ml) | 40 (40,40) | 40 (40, 40) | 2672 (872, 4505) | 7,555 | 709,075 | -14.404 | <0.001 |

Notes: The study subjects consisted of 1,245 people living with HIV (PLWH), with an average age of 43 years (inter-quartile range (*IQR*): 33, 52). The median duration of HIV infection was 7 years (*IQR*: 3 to 13). The mean CD4^+^ T cell counts was 502 cells/μl (*IQR*: 389 cells/μl , 603 cells/μl), and the median HIV viral load was 40 copies/ml (*IQR*: 40, 40). Bh: *Blastocystis hominis.* BMI:Body mass index*.* HIV: Human immunodeficiency virus.

*.*

**Table S3.** The correlation between CD4^+^ T cell counts, HIV viral load, duration of HIV infection, the duration of interruption in highly active antiretroviral therapy in people living with HIV.

| Variable |  | HIV infection  time (year) | Duration of interruption in highly active antiretroviral therapy (month) | CD4^+^ T cell counts (cells/μl) | HIV viral load  (copies/ml) |
| --- | --- | --- | --- | --- | --- |
| HIV infection  time (year) | Correlation Coefficient | 1 | -0.096** | 0.096** | -0.088** |
|  | *P*-value (2-tailed) | . | 0.001 | 0.001 | 0.002 |
|  | *N* | 1245 | 1245 | 1245 | 1245 |
| The duration of interruption in highly active antiretroviral therapy (month) | Correlation Coefficient | -0.096** | 1 | -0.257** | 0.607** |
|  | *P*-value (2-tailed) | 0.001 | - | <0.001 | <0.001 |
|  | *N* | 1245 | 1245 | 1245 | 1245 |
| CD4^+^ T cell counts (cells/μl) | Correlation Coefficient | 0.096** | -0.257** | 1 | -0.557** |
|  | *P*-value (2-tailed) | 0.001 | <0.001 | - | <0.001 |
|  | *N* | 1245 | 1245 | 1245 | 1245 |
| HIV viral load  (copies/ml) | Correlation Coefficient | -0.088** | 0.607** | -0.557** | 1 |
|  | *P*-value (2-tailed) | 0.002 | <0.001 | <0.001 | - |
|  | *N* | 1245 | 1245 | 1245 | 1245 |

Notes: HIV: Human immunodeficiency virus. “**” There is a statistical difference in the correlation between the two variables.” -” The association between the two variables cannot be calculated.

**Table S4.** Multivariable analysis with logistic regression for *Blastocystis hominis i*nfection in persons living with human immunodeficiency virus.

| **Varlables** | **Subgroups** | **Blastocystis  negative *n* (%)** | **Blastocystis  positive *n* (%)** | **Multivariate analysis** | | |
| --- | --- | --- | --- | --- | --- | --- |
|  |  |  |  | **Wald *χ*^2^** | ***P*** | ***OR* (95% *CI*)** |
| CD4^+^T immunological status | No immunosuppression (> 500 cells/μl) | 625 (52.8) | 3 (4.9) | 8.573 | 0.036 | 1 (reference) |
|  | Mild immunosuppression  (350－500 cells/μl) | 396 (33.4) | 15 (24.6) |  |  | 4.665 (1.255－17.332) |
|  | Moderate immunosuppression (200－350 cells/μl) | 83 (7.0) | 21 (34.4) |  |  | 7.245 (1.576－33.304) |
|  | Severe immunosuppression (<= 200 cells/μl) | 80 (6.8) | 22 (36.1) |  |  | 3.902 (0.818－18.602) |
| HIV virological  status | Full virological suppression (< 50 copies/ml) | 968 (81.8) | 4 (6.6) | 23.557 | <0.001 | 1 (reference) |
|  | Low level viraemia (51－999 copies/ml) | 80 (6.8) | 12 (19.7) |  |  | 13.746 (3.946－47.881) |
|  | Virological failure (>1000 copies/ml) | 136 (11.5) | 45 (73.8) |  |  | 24.985 (6.545, 95.372) |
| Treatment interruption | No | 1,087 (91.8) | 27 (44.3) | 12.055 | 0.001 | 1 (reference) |
|  | Yes | 97 (8.2) | 34 (55.7) |  |  | 3.072 (1.630－5.788) |

Notes: The “–” symbol indicates that data could not be calculated. BMI: Body mass index. CIs: Confidence intervals. HIV: Human immunodeficiency virus. OR: Odds ratio.

**Table S5.** Propensity score method was used to explore the association between *Blastocystis hominis* infection and CD4^+^ T immunological status, HIV virological status, and treatment interruption.

| **Variables** | **Subgroups** | **Matching  ratio** | **Caliper** | ***Blastocystis hominis*  Negative *n*** | ***Blastocystis hominis* Positive *n*** | **Wald *χ*^2^** | ***P*** | ***OR* (95% *CI*)** |
| --- | --- | --- | --- | --- | --- | --- | --- | --- |
| CD4^+^ T immunological status^a^ | No immunosuppression (> 500 cells/μl) | 1:1 | 0.10 | 60 | 60 | 15.813 | 0.001 | 1 (reference) |
|  | Mild immunosuppression (350－500 cells/μl) |  |  |  |  |  |  | 9.049 (1.141,71.744) |
|  | Moderate immunosuppression (200－350 cells/μl) |  |  |  |  |  |  | 73.436 (6.302－855.799) |
|  | Severe immunosuppression (<= 200 cells/μl) |  |  |  |  |  |  | 107.450 (8.093－1426.670) |
| CD4^+^ T immunological status^a^ | No immunosuppression (> 500 cells/μl) | 1:1 | 0.20 | 61 | 61 | 17.455 | 0.001 | 1 (reference) |
|  | Mild immunosuppression (350－500 cells/μl) |  |  |  |  |  |  | 5.937 (1.719－20.509) |
|  | Moderate immunosuppression (200－350 cells/μl) |  |  |  |  |  |  | 10.231 (3.052－34.299) |
|  | Severe immunosuppression (<= 200 cells/μl) |  |  |  |  |  |  | 10.718 (3.208－35.809) |
| CD4^+^ T immunological status^a^ | No immunosuppression (> 500 cells/μl) | 1:2 | 0.10 | 56 | 112 | 27.447 | <0.001 | 1 (reference) |
|  | Mild immunosuppression (350－500 cells/μl) |  |  |  |  |  |  | 2.357 (0.624－8.909) |
|  | Moderate immunosuppression (200－350 cells/μl) |  |  |  |  |  |  | 23.333 (5.348－101.811) |
|  | Severe immunosuppression (<= 200 cells/μl) |  |  |  |  |  |  | 27.918 (6.361－122.541) |
| CD4^+^ T immunological status^a^ | No immunosuppression (> 500 cells/μl) | 1:2 | 0.20 | 55 | 110 | 21.417 | <0.001 | 1 (reference) |
|  | Mild immunosuppression (350－500 cells/μl) |  |  |  |  |  |  | 10.579 (1.283－87.266) |
|  | Moderate immunosuppression (200－350 cells/μl) |  |  |  |  |  |  | 108.005 (9.459,1,233.238) |
|  | Severe immunosuppression (<= 200 cells/μl) |  |  |  |  |  |  | 198.363 (16.070－2,448.510) |
| CD4^+^ T immunological status^a^ | No immunosuppression (> 500 cells/μl) | 1:4 | 0.01 | 33 | 132 | 24.974 | <0.001 | 1 (reference) |
|  | Mild immunosuppression (350－500 cells/μl) |  |  |  |  |  |  | 4.929 (1.047－23.209) |
|  | Moderate immunosuppression (200－350 cells/μl) |  |  |  |  |  |  | 17.250 (3.727－79.837) |
|  | Severe immunosuppression (<= 200 cells/μl) |  |  |  |  |  |  | 21.955 (4.990－96.599) |
| CD4^+^ T immunological status^a^ | No immunosuppression (> 500 cells/μl) | 1:4 | 0.02 | 42 | 168 | 29.575 | <0.001 | 1 (reference) |
|  | Mild immunosuppression (350－500 cells/μl) |  |  |  |  |  |  | 6.385 (1.781－22.887) |
|  | Moderate immunosuppression (200－350 cells/μl) |  |  |  |  |  |  | 14.813 (4.133－53.097) |
|  | Severe immunosuppression (<= 200 cells/μl) |  |  |  |  |  |  | 22.013 (6.451－75.114) |
| CD4^+^ T immunological status^a^ | No immunosuppression (> 500 cells/μl) | 1:4 | 0.10 | 47 | 188 | 42.064 | <0.001 | 1 (rReference) |
|  | Mild immunosuppression (350－500 cells/μl) |  |  |  |  |  |  | 4.250 (1.170－15.443) |
|  | Moderate immunosuppression (200－350 cells/μl) |  |  |  |  |  |  | 19.040 (5.472－66.254) |
|  | Severe immunosuppression (<= 200 cells/μl) |  |  |  |  |  |  | 24.286 (7.217－81.727) |
| CD4^+^ T immunological status^a^ | No immunosuppression (> 500 cells/μl) | 1:4 | 0.20 | 49 | 196 | 37.331 | <0.001 | 1 (reference) |
|  | Mild immunosuppression (350－500 cells/μl) |  |  |  |  |  |  | 4.200 (1.137－15.521) |
|  | Moderate immunosuppression (200－350 cells/μl) |  |  |  |  |  |  | 28.252 (7.109－112.277) |
|  | Severe immunosuppression (<= 200 cells/μl) |  |  |  |  |  |  | 30.071 (7.883－114.710) |
| HIV virological status ^b^ | Full virological suppression (< 50 copies/ml) | 1:1 | 0.10 | 60 | 60 | 20.860 | <0.001 | 1 (reference) |
|  | Low level viraemia (51－999 copies/ml) |  |  |  |  |  |  | 7.895 (2.546－24.478) |
|  | Virological failure (>1,000 copies/ml) |  |  |  |  |  |  | 10.784 (3.875－30.014) |
| HIV virological status ^b^ | Full virological suppression(<50 copies/mL) | 1:1 | 0.20 | 61 | 61 | 13.492 | <0.001 | 1 (reference) |
|  | Low level viraemia(51-999 copies/mL) |  |  |  |  |  |  | 36.987( 4.013－340.904) |
|  | Virological failure(>1000 copies/mL) |  |  |  |  |  |  | 42.071 (5.685－311.327) |
| HIV virological status ^b^ | Full virological suppression(<50 copies/mL) | 1:2 | 0.10 | 55 | 110 | 23.955 | <0.001 | 1 (reference) |
|  | Low level viraemia(51-999 copies/mL) |  |  |  |  |  |  | 19.160 (3.394－108.158) |
|  | Virological failure(>1000 copies/mL) |  |  |  |  |  |  | 39.253 (9.021－170.807) |
| HIV virological status ^b^ | Full virological suppression (< 50 copies/ml) | 1:2 | 0.20 | 56 | 112 | 20.825 | <0.001 | 1 (reference) |
|  | Low level viraemia (51－999 copies/ml) |  |  |  |  |  |  | 26.451 (3.270－213.962) |
|  | Virological failure (>1,000 copies/ml) |  |  |  |  |  |  | 173.267( 18.694－1605.983) |
| HIV virological status ^b^ | Full virological suppression (< 50 copies/ml) | 1:4 | 0.01 | 33 | 132 | 18.976 | <0.001 | 1 (Reference) |
|  | Low level viraemia (51－999 copies/ml) |  |  |  |  |  |  | 45.325 (5.490－374.198) |
|  | Virological failure (>1,000 copies/ml) |  |  |  |  |  |  | 96.162 (12.247－755.071) |
| HIV virological status ^b^ | Full virological suppression (< 50 copies/ml) | 1:4 | 0.02 | 42 | 168 | 23.348 | <0.001 | 1 (reference) |
|  | Low level viraemia (51－999 copies/ml) |  |  |  |  |  |  | 36.532 (4.519－295.310) |
|  | Virological failure (>1000 copies/ml) |  |  |  |  |  |  | 263.515 (27.237－2549.478) |
| HIV virological status ^b^ | Full virological suppression (< 50 copies/ml) | 1:4 | 0.10 | 47 | 188 | 21.813 | <0.001 | 1 (reference) |
|  | Low level viraemia (51－999 copies/ml) |  |  |  |  |  |  | 84.668 (9.816－730.277) |
|  | Virological failure (>1000 copies/ml) |  |  |  |  |  |  | 119.257 (16.036－886.911) |
| HIV virological status ^b^ | Full virological suppression (< 50 copies/ml) | 1:4 | 0.20 | 48 | 192 | 22.207 | <0.001 | 1 (reference) |
|  | Low level viraemia (51－999 copies/ml) |  |  |  |  |  |  | 59.989 (7.108－506.260) |
|  | Virological failure (>1000 copies/ml) |  |  |  |  |  |  | 118.151 (15.851－880.673) |
| Treatment interruption ^c^ | Treatment interruption (No) | 1:1 | 0.10 | 60 | 60 | 13.001 | <0.001 | 1 (reference) |
|  | Treatment interruption (Yes) |  |  |  |  |  |  | 14.000 (3.335－58.768) |
| Treatment interruption ^c^ | Treatment interruption (No) | 1:1 | 0.20 | 61 | 61 | 15.368 | <0.001 | 1 (reference) |
|  | Treatment interruption (Yes) |  |  |  |  |  |  | 10.667 (3.266－34.833) |
| Treatment interruption ^c^ | Treatment interruption (No) | 1:2 | 0.10 | 56 | 112 | 20.831 | <0.001 | 1 (reference) |
|  | Treatment interruption (Yes) |  |  |  |  |  |  | 6.264 (2.849－13.772) |
| Treatment interruption ^c^ | Treatment interruption (No) | 1:2 | 0.20 | 56 | 112 | 22.130 | <0.001 | 1 (reference) |
|  | Treatment interruption (Yes) |  |  |  |  |  |  | 12.476 (4.359－35.707) |
| Treatment interruption ^c^ | Treatment interruption (No) | 1:4 | 0.01 | 33 | 132 | 20.732 | <0.001 | 1 (reference) |
|  | Treatment interruption (Yes) |  |  |  |  |  |  | 30.426 (6.995－132.349) |
| Treatment interruption ^c^ | Treatment interruption (No) | 1:4 | 0.02 | 42 | 168 | 27.781 | <0.001 | 1 (reference) |
|  | Treatment interruption (Yes) |  |  |  |  |  |  | 14.191 (5.292－38.055) |
| Treatment interruption ^c^ | Treatment interruption (No) | 1:4 | 0.10 | 47 | 188 | 34.255 | <0.001 | 1 (reference) |
|  | Treatment interruption (Yes) |  |  |  |  |  |  | 14.536 (5.932－35.622) |
| Treatment interruption ^c^ | Treatment interruption (No) | 1:4 | 0.20 | 49 | 196 | 31.658 | <0.001 | 1 (reference) |
|  | Treatment interruption (Yes) |  |  |  |  |  |  | 13.264 (5.390－32.639) |

Notes: a: adjusted for age (>= 60 years vs < 60 years), body mass index (< 18.5, 18.5－24.9, > 25.0), family members (< 3 individuals vs >= 3 individuals), gender, residence, region, occupation, nationality, marital status, educational level, toilet type, water supply, drinking water, livestock, pet, HIV infection route, newly infection, HIV VL, treatment interruption. b: adjusted for age (>= 60 years vs < 60 years), body mass index (< 18.5, 18.5－24.9, >= 25.0 kg/m^2^), family members (< 3 individuals vs >= 3 individuals), gender, residence, region, occupation, nationality, marital status, educational level, toilet type, water supply, drinking water, livestock, pet, HIV infection route, newly infection, CD4^+^ T cell counts, duration of treatment interruption in HARRT. c: adjusted for age (>= 60 years vs < 60 years), body mass index (< 18.5, 18.5－24.9, >= 25.0 kg/m^2^), family members (<3 individuals vs >= 3 individuals), gender, residence, region, occupation, nationality, marital status, educational level, toilet type, water supply, drinking water, livestock, pet, HIV infection route, newly infection, CD4^+^ T cell counts, HIV VL. CIs: Confidence intervals. HAART: Highly active antiretroviral therapy. HIV: Human immunodeficiency virus. OR: Odds ratio. VL: Viral load.

**Table S6.** The study employs propensity scores to investigate the association between *Blastocystis hominis* infection and CD4^+^ T cell counts (cells/μl), HIV viral load (copies/ml), and the duration of interruption in antiretroviral therapy (month).

| **Variables** | **Matching**  **ratio** | **Caliper** | **Bh negative**  ***n*** | **Bh**  **positive**  ***n*** | **Wald χ ^2^** | ***P*** | ***OR* (95% *CI*)** |
| --- | --- | --- | --- | --- | --- | --- | --- |
| CD4 ^+^ T counts (cells/μl)^a^ | 1:1 | 0.01 | 55 | 55 | 10.667 | <0.001 | 0.998 (0.996－0.999) |
|  |  |  |  |  |  |  |  |
|  |  |  |  |  |  |  |  |
|  |  |  |  |  |  |  |  |
| CD4^+^ T counts (cells/μl)^a^ | 1:1 | 0.02 | 57 | 57 | 15.353 | <0.001 | 0.997 (0.996－0.999) |
|  |  |  |  |  |  |  |  |
|  |  |  |  |  |  |  |  |
|  |  |  |  |  |  |  |  |
| CD4^+^ T counts (cells/μl)^a^ | 1:1 | 0.10 | 60 | 60 | 15.217 | <0.001 | 0.993 (0.990－0.997) |
|  |  |  |  |  |  |  |  |
|  |  |  |  |  |  |  |  |
|  |  |  |  |  |  |  |  |
| CD4^+^ T counts (cells/μl)^a^ | 1:1 | 0.20 | 61 | 61 | 15.605 | <0.001 | 0.997(0.996－0.999) |
|  |  |  |  |  |  |  |  |
|  |  |  |  |  |  |  |  |
|  |  |  |  |  |  |  |  |
| CD4^+^T counts (cells/μl)^a^ | 1:2 | 0.01 | 44 | 88 | 16.676 | <0.001 | 0.995 (0.992－0.997) |
|  |  |  |  |  |  |  |  |
|  |  |  |  |  |  |  |  |
|  |  |  |  |  |  |  |  |
| CD4^+^ T counts (cells/μl)^a^ | 1:2 | 0.02 | 49 | 98 | 21.392 | <0.001 | 0.996 (0.995－0.998) |
|  |  |  |  |  |  |  |  |
|  |  |  |  |  |  |  |  |
|  |  |  |  |  |  |  |  |
| CD4^+^ T counts (cells/μl)^a^ | 1:2 | 0.10 | 56 | 112 | 22.051 | <0.001 | 0.994 (0.992－0.997) |
|  |  |  |  |  |  |  |  |
|  |  |  |  |  |  |  |  |
|  |  |  |  |  |  |  |  |
| CD4^+^ T counts (cells/μl)^a^ | 1:2 | 0.20 | 55 | 110 | 23.483 | <0.001 | 0.992 (0.989－0.995) |
|  |  |  |  |  |  |  |  |
|  |  |  |  |  |  |  |  |
|  |  |  |  |  |  |  |  |
| CD 4 ^+^ T counts (cells/μl)^a^ | 1:4 | 0.01 | 33 | 132 | 21.436 | <0.001 | 0.992 (0.989－0.995) |
|  |  |  |  |  |  |  |  |
|  |  |  |  |  |  |  |  |
|  |  |  |  |  |  |  |  |
| CD4^+^ T counts (cells/μl)^a^ | 1:4 | 0.02 | 42 | 168 | 29.216 | <0.001 | 0.995 (0.994－0.997) |
|  |  |  |  |  |  |  |  |
|  |  |  |  |  |  |  |  |
|  |  |  |  |  |  |  |  |
| CD4^+^ T counts (cells/μl)^a^ | 1:4 | 0.10 | 47 | 188 | 40.159 | <0.001 | 0.995 (0.993－0.996) |
|  |  |  |  |  |  |  |  |
|  |  |  |  |  |  |  |  |
|  |  |  |  |  |  |  |  |
| CD4^+^ T counts (cells/μl)^a^ | 1:4 | 0.20 | 49 | 196 | 30.294 | <0.001 | 0.994 (0.992,0.996) |
|  |  |  |  |  |  |  |  |
|  |  |  |  |  |  |  |  |
|  |  |  |  |  |  |  |  |
| HIV viral load (copies/ml)^b^ | 1:1 | 0.01 | 55 | 55 | 10.305 | <0.001 | 1.0003 (1.001－1.0005) |
|  |  |  |  |  |  |  |  |
|  |  |  |  |  |  |  |  |
| HIV viral load (copies/ml)^b^ | 1:1 | 0.02 | 57 | 57 | 11.427 | <0.001 | 1.0004 (1.0001－1.0009) |
|  |  |  |  |  |  |  |  |
|  |  |  |  |  |  |  |  |
| HIV viral load (copies/ml)^b^ | 1:1 | 0.10 | 60 | 60 | 13.101 | <0.001 | 1.0001 (1.00001－1.0010) |
|  |  |  |  |  |  |  |  |
|  |  |  |  |  |  |  |  |
| HIV viral load (copies/ml)^b^ | 1:1 | 0.20 | 61 | 61 | 10.401 | <0.001 | 1.0003 (1.0002－1.0004) |
|  |  |  |  |  |  |  |  |
|  |  |  |  |  |  |  |  |
| HIV viral load (copies/ml)^b^ | 1:2 | 0.01 | 47 | 94 | 24.467 | <0.001 | 1.00015 (1.00009－1.0002) |
|  |  |  |  |  |  |  |  |
|  |  |  |  |  |  |  |  |
| HIV viral load (copies/ml)^b^ | 1:2 | 0.02 | 49 | 98 | 25.577 | <0.001 | 1.00015 (1.00009－1.0002) |
|  |  |  |  |  |  |  |  |
|  |  |  |  |  |  |  |  |
| HIV viral load(copies/ml)^b^ | 1:2 | 0.10 | 55 | 110 | 21.421 | <0.001 | 1.0001 (1.0003－1.0009) |
|  |  |  |  |  |  |  |  |
|  |  |  |  |  |  |  |  |
| HIV viral load (copies/ml)^b^ | 1:2 | 0.20 | 56 | 112 | 11.297 | <0.001 | 1.0023 (1.00099－1.0043) |
|  |  |  |  |  |  |  |  |
|  |  |  |  |  |  |  |  |
| HIV viral load (copies/ml)^b^ | 1:4 | 0.01 | 33 | 132 | 8.261 | <0.001 | 1.0005 (1.0003－1.0008) |
|  |  |  |  |  |  |  |  |
|  |  |  |  |  |  |  |  |
| HIV viral load (copies/ml)^b^ | 1:4 | 0.02 | 42 | 168 | 20.521 | <0.001 | 1.0011 (1.0006－1.0015) |
|  |  |  |  |  |  |  |  |
|  |  |  |  |  |  |  |  |
| HIV viral load (copies/mL)^b^ | 1:4 | 0.10 | 47 | 188 | 28.685 | <0.001 | 1.0005 (1.0003－1.0008) |
|  |  |  |  |  |  |  |  |
|  |  |  |  |  |  |  |  |
| HIV viral load (copies/mL)^b^ | 1:4 | 0.20 | 48 | 192 | 28.255 | <0.001 | 1.0005 (1.0003－1.0007) |
|  |  |  |  |  |  |  |  |
|  |  |  |  |  |  |  |  |
| Duration of interruption in highly active antiretroviral therapy (month)^c^ | 1:1 | 0.01 | 55 | 55 | 9.066 | 0.003 | 1.628 (1.185－2.236) |
|  |  |  |  |  |  |  |  |
| Duration of interruption in highly active antiretroviral therapy (month)^c^ | 1:1 | 0.02 | 57 | 57 | 8.996 | 0.003 | 1.701 (1.202－2.407) |
|  |  |  |  |  |  |  |  |
| Duration of interruption in highly active antiretroviral therapy (month)^c^ | 1:1 | 0.10 | 60 | 60 | 10.405 | 0.001 | 1.797 (1.258－2.565) |
|  |  |  |  |  |  |  |  |
| Duration of interruption in highly active antiretroviral therapy (month)^c^ | 1:1 | 0.20 | 61 | 61 | 10.945 | 0.001 | 1.421 (1.154－1.749) |
|  |  |  |  |  |  |  |  |
| Duration of interruption in highly active antiretroviral therapy(month)^c^ | 1:2 | 0.01 | 46 | 92 | 9.982 | 0.002 | 2.601 (1.437－4.705) |
|  |  |  |  |  |  |  |  |
| Duration of interruptions in highly active antiretroviral therapy (month)^c^ | 1:2 | 0.02 | 49 | 98 | 13.259 | <0.001 | 1.941 (1.358－2.773) |
|  |  |  |  |  |  |  |  |
| Duration of interruption in highly active antiretroviral therapy (month)^c^ | 1:2 | 0.10 | 56 | 112 | 19.168 | <0.001 | 1.617 (1.304－2.006) |
|  |  |  |  |  |  |  |  |
| Duration of interruption in highly active antiretroviral therapy (month)^c^ | 1:2 | 0.20 | 56 | 112 | 18.216 | <0.001 | 1.614 (1.295－2.011) |
|  |  |  |  |  |  |  |  |
| Duration of interruption in highly active antiretroviral therapy (month)^c^ | 1:4 | 0.01 | 33 | 132 | 9.106 | 0.003 | 3.812 (1.598－9.089) |
|  |  |  |  |  |  |  |  |
| Duration of interruption in highly active antiretroviral therapy (month)^c^ | 1:4 | 0.02 | 42 | 168 | 12.759 | <0.001 | 2.316 (1.461－3.672) |
|  |  |  |  |  |  |  |  |
| Duration of interruption in highly active antiretroviral therapy (month)^c^ | 1:4 | 0.10 | 47 | 188 | 22.435 | <0.001 | 1.563 (1.299－1.880) |
|  |  |  |  |  |  |  |  |
| Duration of interruption in highly active antiretroviral therapy (month)^c^ | 1:4 | 0.20 | 49 | 196 | 22.126 | <0.001 | 1.713 (1.369－2.144) |
|  |  |  |  |  |  |  |  |

Notes: a: adjusted for age, BMI, family members, gender, residence, region, occupation, nationality, marital status, educational level, toilet type, water supply, drinking water, livestock, pet, HIV infection route, newly infection, HIV viral load (copies/ml), duration of interruption in highly active antiretroviral therapy (month). b: adjusted for age, BMI, family members, gender, residence, region, occupation, nationality. marital status, educational level, toilet type, water supply, drinking water, livestock, pet, HIV infection route, newly infection, HIV viral load (copies/ml), duration of interruption in highly active antiretroviral therapy (month). c: adjusted for age, BMI, family members, gender, residence, region, occupation, nationality, marital status, educational level, toilet type, water supply, drinking water, livestock, pet, HIV infection route, newly infection, CD4^+^ T cell counts, HIV viral load. Bh: *Blastocystis hominis.* BMI: Body mass index. HIV: Human immunodeficiency virus.

**Table S7**. Univariate linear models and nonlinear models was used to investigate the relationship between *Blastocystis hominis* infection and CD4^+^ T cell counts (cells/μl), HIV viral load (copies/ml), and the duration of interruption in highly active antiretroviral therapy (month).

| Variables | Model | AIC | *P -*value (Compare.test) | *P-*value (Nonlinear) |
| --- | --- | --- | --- | --- |
| CD4^+^ T cell counts (cells/μl) | Restricted cubic splines | 393.512450472034 | 2.81584149327552e-05 | 3.51319436764364e-07 |
| CD4^+^T cell counts (cells/μl) | Logistic model | 410.467779104348 |  |  |
| HIV viral load (copies/ml) | Restricted cubic splines | 372.451795389127 | 1.36112860982252e-08 | 6.33245622605472e-09 |
| HIV viral load(copies/ml) | Logistic model | 402.693845494199 |  |  |
| Duration of interruption in highly active antiretroviral therapy (month) | Restricted cubic splines | 398.830568152767 | 1.35986026661428e-05 | 8.49118034285112e-06 |
| Duration of interruption in highly active antiretroviral therapy (month) | Logistic model | 415.755192466055 |  |  |
| Age | Restricted cubic splines | 492.780381511256 | 0.546382290917289 | 0.569551880007065 |
| Age | Logistic model | 489.989254274704 |  |  |
| BMI | Restricted cubic splines | 493.786168143632 | 0.7201342996598 | 0.729466896195805 |
| BMI | Logistic model | 490.442803257754 |  |  |
| Family members | Restricted cubic splines | 492.610882732569 | 0.464684515097573 | 0.473260394394229 |
| Family members | Logistic model | 490.143675864189 |  |  |
| HIV infection time | Restricted cubic splines | 487.151638183407 | 0.319564551513716 | 0.316639358019634 |
| HIV infection time | Logistic model | 485.433230156217 |  |  |

Notes:　For CD4^+^ T cell counts (cells/μl), HIV viral load (copies/ml) and Duration of interruption in highly active antiretroviral therapy (month), The *P*-values of the nonlinear model are all less than 0.001. AIC: Akaike information criterion. BMI: Body mass index. HIV: Human immunodeficiency virus.

**Table S8.** E-value analyses were employed to examine the strength and robustness of associations between *Blastocystis hominis* infection and CD4^+^ T immunological status－HIV virological status－as well as treatment interruption.

| **Variables** | **Contrast** | **B** | **S.E.** | **Wald χ^2^** | **df** | ***P*-value** | **OR (95% *CI*)** | **Assuming the association**  **between unmeasured confounding and *Blastocystis hominis*** | **Assuming the association**  **between unmeasured confounding and influencing factors** | **Coefficient** | ***RR* true**  **(95% *CI*)** | **E-value** |
| --- | --- | --- | --- | --- | --- | --- | --- | --- | --- | --- | --- | --- |
| CD4^+^ T immunological  status |  |  |  | 9.429 | 3 | 0.024 |  | 5 | 5 | 2.778 | － | － |
| Mild immunosuppression  (350－500 cells/μl) | No immunosuppression  (> 500 cells/μl) | 1.607 | 0.693 | 5.374 | 1 | 0.02 | 4.988  (1.282－19.405) | 5 | 5 | 2.778 | 1.796  (1.000－6.989) | 9.448 |
| Moderate immunosuppression  (200－350 cells/μl) | No immunosuppression  (> 500 cells/μl) | 2.187 | 0.806 | 7.368 | 1 | 0.007 | 8.904  (1.836－43.177) | 5 | 5 | 2.778 | 3.205  (1.000－15.544) | 17.293 |
| Severe immunosuppression  (<= 200 cells/μl) | No immunosuppression  (> 500 cells/μl) | 1.407 | 0.819 | 2.951 | 1 | 0.086 | 4.083  (0.820－20.322) | 5 | 5 | 2.778 | 1.470  (1.000－7.316) | 7.631 |
| HIV virological status |  |  |  | 23.797 | 2 | <0.001 |  | 5 | 5 | 2.778 | - | - |
| Low level viraemia  (51－999 copies/ml) | Full virological suppression  (< 50 copies/ml) | 2.598 | 0.652 | 15.856 | 1 | <0.001 | 13.435  (3.741－48.259) | 5 | 5 | 2.778 | 4.837  (1.346－17.373) | 26.361 |
| Virological failure  (> 1,000 copies/ml) | Full virological suppression  (< 50 copies/ml) | 3.346 | 0.701 | 22.767 | 1 | <0.001 | 28.392  (7.182－112.231) | 5 | 5 | 2.778 | 10.221  (2.586－40.403) | 56.279 |
| HIV treatment interruption (Yes) | HIV treatment interruption (No) | 1.385 | 0.365 | 14.414 | 1 | <0.001 | 3.993  (1.954－8.161) | 5 | 5 | 2.778 | 1.437  (1.000－2.934) | 7.451 |

Notes: B: beta. CIs:Confidence intervals. HIV: Human immunodeficiency virus. OR: Odds ratio. RR: Relative risk. SE: standard error. The “–” symbol indicates that data could not be calculated.

**Table S9.** The least absolute shrinkage and selection operator (lasso) logistic regression was conducted to explore the risk factors associated with *Blastocystis hominis* infection in individuals living with human immunodeficiency virus/Acquired Immune Deficiency Syndrome.

| Variable type | Parameter | Degree of freedom | Estimate |
| --- | --- | --- | --- |
| Quantitative variables ^a^ | Intercept | 1 | -1.024501 |
|  | CD4^+^ T cell immunological status 1 | 1 | -0.376635 |
|  | CD4^+^ T cell immunological status 2 | 1 | -0.014595 |
|  | CD4^+^ T cell immunological status 3 | 1 | 0.265529 |
|  | HIV virological status 1 | 1 | -2.43199 |
|  | HIV virological status 2 | 1 | -0.380128 |
|  | HIV treatment interruption | 1 | -0.782724 |
| Qualitative variables ^b^ | Intercept | 1 | -2.965802 |
|  | CD4^+^ T cell counts | 1 | -0.001255 |
|  | HIV VL | 1 | 0.00038 |

Notes: a: The dependent variable is *Blastocystis hominis* infection, the independent variable including age (categorical variable: >= 60 years vs < 60 years), BMI (categorical variable, low/normal/high), gender, residence, region, occupation, nationality, marital status, education level, toilet type, water supply, drinking water, livestock, pet, HIV infection route, CD4^+^ T immunological status, HIV virological status, treatment interruption, newly infection, b: The dependent variable is *Blastocystis hominis* infection, the independent variable including gender, residence, region, occupation, nationality, marital status, education level, toilet type, water supply, drinking water, livestock, pet, HIV infection route, age, BMI, family members, HIV infection time, CD4^+^ T cell counts, HIV VL, Duration of interruption in highly active antiretroviral therapy. BMI: Body mass index. HIV: Human immunodeficiency virus.

**Table S10.**  Model evaluation indicators for these four algorithms (ANN,GBM, RF and XGBOOST)

| Models | Data sets | True  (diagnose) | Predicted  Positive | Predicted  Negative | Total 1 | Total 2 | Accuracy  (ACC) | Precision  (PPV) | Sensitivity  （TPR, recall） | Specificity  (TNR) | F1 score |
| --- | --- | --- | --- | --- | --- | --- | --- | --- | --- | --- | --- |
| xgboost^a^ | Training set | Positive (true) | 41 | 1 | 42 | 875 | 81.83 | 79.40 | 97.62 | 81.03 | 0.88 |
|  |  | Negative (true) | 158 | 675 | 833 |  |  |  |  |  |  |
|  | Testing set | Positive (true) | 16 | 3 | 19 | 370 | 87.30 | 73.33 | 84.21 | 87.46 | 0.78 |
|  |  | Negative (true) | 44 | 307 | 351 |  |  |  |  |  |  |
| RF^a^ | Training set | Positive (true) | 41 | 1 | 42 | 875 | 84.23 | 76.97 | 97.62 | 83.55 | 0.86 |
|  |  | Negative (true) | 137 | 696 | 833 |  |  |  |  |  |  |
|  | Testing set | Positive (true) | 15 | 4 | 19 | 370 | 88.38 | 72.22 | 78.95 | 88.89 | 0.75 |
|  |  | Negative (true) | 39 | 312 | 351 |  |  |  |  |  |  |
| GBM^a^ | Training set | Positive (true) | 41 | 1 | 42 | 875 | 80.57 | 80.48 | 97.62 | 79.71 | 0.88 |
|  |  | Negative (true) | 169 | 664 | 833 |  |  |  |  |  |  |
|  | Testing set | Positive (true) | 16 | 3 | 19 | 360 | 88.89 | 69.81 | 84.21 | 89.15 | 0.76 |
|  |  | Negative (true) | 37 | 304 | 341 |  |  |  |  |  |  |
| ANN^a^ | Training set | Positive (true) | 38 | 4 | 42 | 875 | 84.80 | 77.25 | 90.48 | 84.51 | 0.83 |
|  |  | Negative (true) | 129 | 704 | 833 |  |  |  |  |  |  |
|  | Testing set | Positive (true) | 14 | 5 | 19 | 370 | 89.19 | 71.43 | 73.68 | 90.03 | 0.73 |
|  |  | Negative (true) | 35 | 316 | 351 |  |  |  |  |  |  |
| xgboost^b^ | Training set | Positive (true) | 35 | 5 | 40 | 870 | 81.72 | 81.48 | 87.50 | 81.45 | 0.84 |
|  |  | Negative (true) | 154 | 676 | 830 |  |  |  |  |  |  |
|  | Testing set | Positive (true) | 14 | 4 | 18 | 370 | 85.41 | 78.13 | 77.78 | 85.80 | 0.78 |
|  |  | Negative (true) | 50 | 302 | 352 |  |  |  |  |  |  |
| RF^b^ | Training set | Positive (true) | 39 | 2 | 41 | 870 | 85.29 | 76.36 | 95.12 | 84.80 | 0.85 |
|  |  | Negative (true) | 126 | 703 | 829 |  |  |  |  |  |  |
|  | Testing set | Positive (true) | 11 | 3 | 14 | 370 | 87.03 | 80.36 | 78.57 | 87.36 | 0.79 |
|  |  | Negative (true) | 45 | 311 | 356 |  |  |  |  |  |  |
| GMB^b^ | Training set | Positive (true) | 35 | 4 | 39 | 871 | 86.57 | 76.35 | 89.74 | 86.42 | 0.83 |
|  |  | Negative (true) | 113 | 719 | 832 |  |  |  |  |  |  |
|  | Testing set | Positive (true) | 15 | 3 | 18 | 370 | 71.62 | 87.18 | 83.33 | 71.02 | 0.85 |
|  |  | Negative (true) | 102 | 250 | 352 |  |  |  |  |  |  |
| ANN^b^ | Training set | Positive (true) | 33 | 3 | 36 | 870 | 85.86 | 78.43 | 91.67 | 85.61 | 0.85 |
|  |  | Negative (true) | 120 | 714 | 834 |  |  |  |  |  |  |
|  | Testing set | Positive (true) | 12 | 3 | 15 | 370 | 90.27 | 73.33 | 80.00 | 90.70 | 0.77 |
|  |  | Negative (true) | 33 | 322 | 355 |  |  |  |  |  |  |

Notes: a: The dependent variable is *Blastocystis hominis* infection, the independent variable including age (categorical variable), BMI (categorical variable), gender, residence, region, occupation, nationality, marital status, education level, toilet type, water supply, drinking water, livestock, pet, HIV infection route, CD4^+^ T immunological status, HIV virological status, treatment interruption, newly infection, b: The dependent variable is *Blastocystis hominis* infection, the independent variable including age, gender, residence, region, occupation, nationality, marital status, education level, toilet type, water supply, drinking water, livestock, pet, HIV infection route, bmi, family members, HIV infection time, CD4^+^ T cell counts, HIV viral load, duration of interruptions in highly active antiretroviral therapy. ANN: Artificial neural network. BMI: Body mass index. HIV: Human immunodeficiency virus. GBM: Gradient boosting machine. RF: Random forest. XGBOOST: EXtreme gradient boosting.


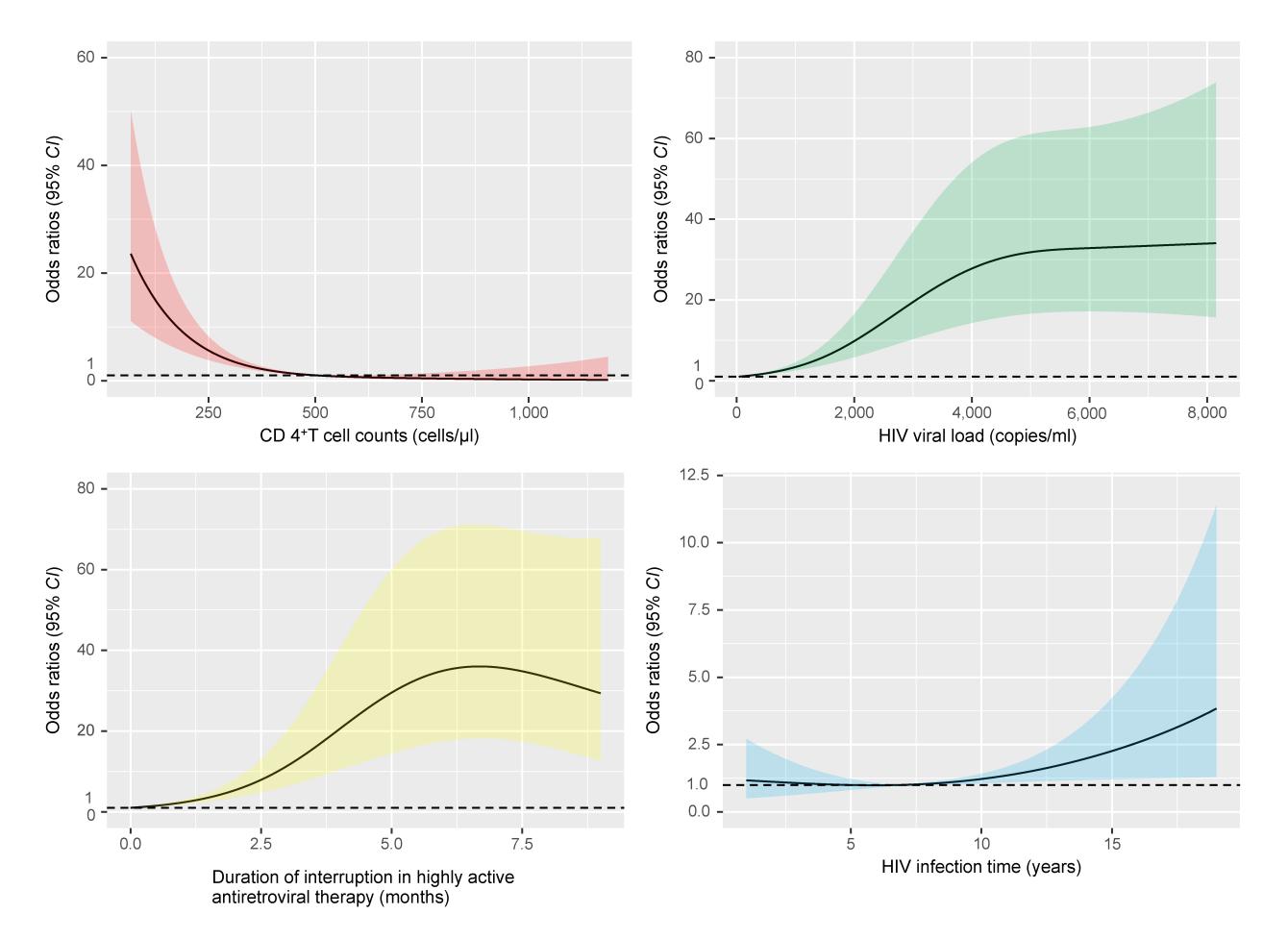


**Fig. S1** Restricted cubic spline (RCS, three knots, 10^th^, 50^th^ and 90^th^ percentiles) was applied to explore the association between *Blastocystis hominis* infection risk and HIV infection time (year), CD4^+^ T counts, HIV viral load and duration of interruption in highly active antiretroviral therapy (month). In each single model, there are no other adjustment factors. HIV: Human immunodeficiency virus.


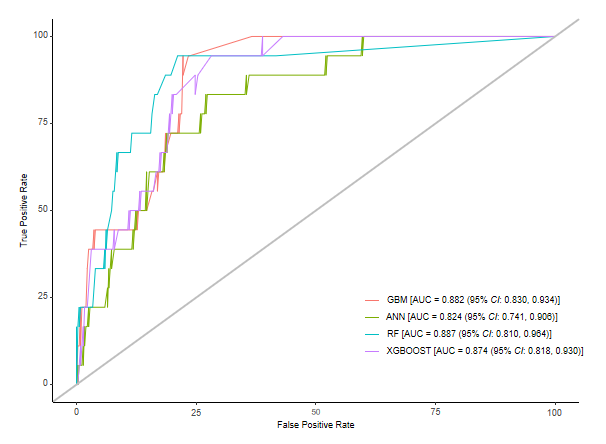


**Fig. S2** The four algorithms (ANN,GBM, RF and XGBOOST) were applied for screening of risk factors associated with *Blastocystis hominis* infection in persons living with HIV (area under curve. Categorical variable: CD4^+^ T cell immunological status, HIV virological status, treatment interruption). GBM: Gradient boosting machine. ANN: Artificial neural network. RF: Random forest. HIV: Human immunodeficiency virus. XGBOOST: EXtreme gradient boosting.


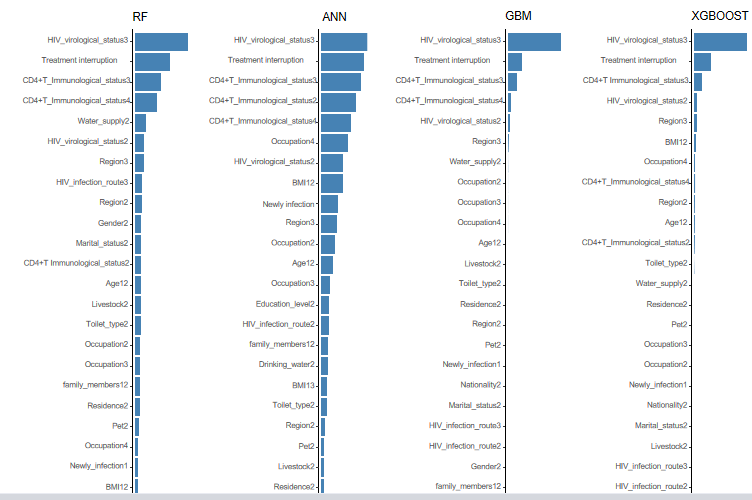


**Fig. S3** The four algorithms (ANN, GBM, RF and XGBOOST) were adopted for screening of the risk factors associated with *Blastocystis hominis* infection in person living with HIV (feature selection. Categorical variable: CD4^+^ T cell Immunological status, HIV virological status, HIV treatment interruption (Yes/no)). ANN: Artificial neural network. GBM: Gradient boosting machine. HIV: Human immunodeficiency virus. RF: Random forest. XGBOOST: EXtreme gradient boosting.


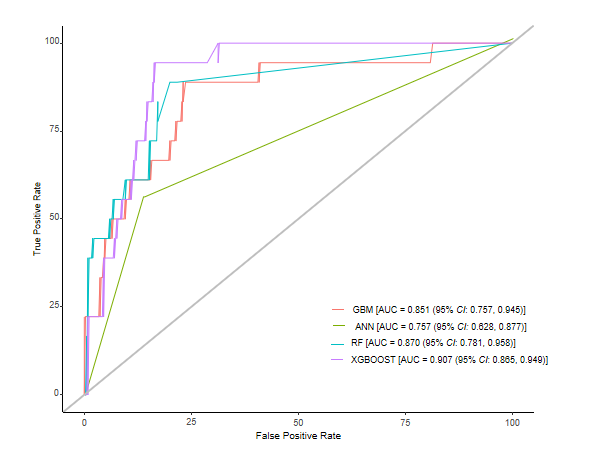


**Fig.S4** The four algorithms (ANN, GBM, RF and XGBOOST) were applied for demonstrating the all risk factors associated with *Blastocystis hominis infection* in person living with HIV (area under curve. Continuous variable: CD4^+^ T cell counts (cells/μl), HIV viral load (copies/ml), and duration of interruptions in highly active antiretroviral therapy (month)). ANN: Artificial neural network. GBM: Gradient boosting machine. HIV: Human immunodeficiency virus. RF: Random forest. XGBOOST: EXtreme gradient boosting.


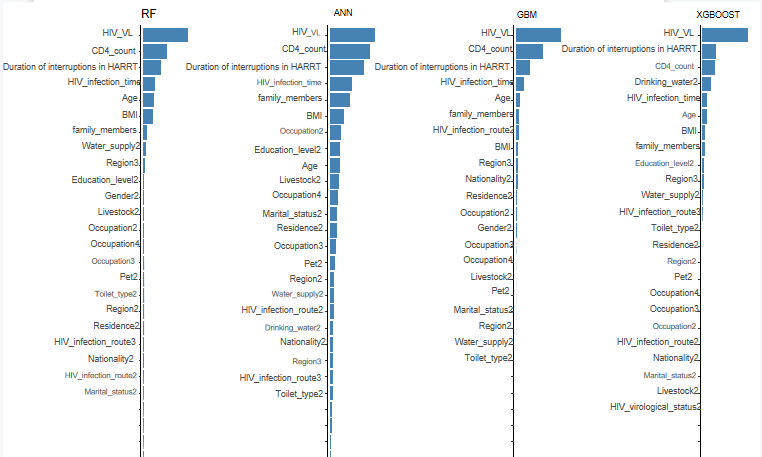


**Fig. S5** The four algorithms (ANN, GBM, RF and XGBOOST) were adopted for indicating the all risk factors associated with *Blastocystis hominis* infection in persons living with HIV (feature selection. Continuous variable: CD4^+^ T cell counts (cells/μl), HIV viral load (copies/ml), and the duration of interruption in highly active antiretroviral therapy). ANN: Artificial neural network. GBM: Gradient boosting machine. HIV: Human immunodeficiency virus. RF: Random forest. XGBOOST: EXtreme gradient boosting.
